# A numerical investigation of the effect of micro vortex generators on film cooling

**DOI:** 10.1038/s41598-025-29054-7

**Published:** 2025-11-23

**Authors:** Mana Suwari, Ehsan Roohi

**Affiliations:** 1 Department of Mechanical Engineering, Ferdowsi University of Mashhad, P.O.B. 91775-1111, Mashhad, Iran; 2 Mechanical and Industrial Engineering, University of Massachusetts Amherst, 160 Governors Dr., Amherst, MA 01003 USA

**Keywords:** Film cooling effectiveness, Micro vortex generator (MVG), Stator blade, Surface temperature, Turbulence kinetic energy (TKE), Numerical investigation, Engineering, Aerospace engineering, Mechanical engineering

## Abstract

Film cooling is a key technology for protecting turbine blades from high thermal loads, directly influencing component durability, efficiency, and operational safety. Micro vortex generators (MVGs) offer a passive approach to enhance near-wall mixing, suppress cooling jet lift-off, and stabilize the coolant film, enabling more uniform and effective surface cooling. Despite their widespread use, the detailed influence of MVG geometry, axial placement, and tip angle on aerodynamic and thermal performance remains insufficiently understood. This study examines MVG height (1.5 to 5.5 mm), axial location (− 7.5 to + 7.5 mm relative to the cooling hole), and tip angle (tested within ± 5° of the baseline design) on film cooling over a stator blade. Aerodynamic and thermal effects are evaluated using turbulence kinetic energy (TKE), surface pressure distributions, temperature, film cooling effectiveness, enthalpy, stagnation density, cfRe (friction Reynolds number), Nusselt number, adiabatic film cooling effectiveness, and pressure-loss measurements. Heights below 1.5 mm fail to generate coherent vortices, acting mainly as surface roughness, while heights above 5.5 mm induce local separation, vortex breakdown, and increased pressure loss, reducing overall cooling. Heights of 2.5–4.0 mm produce stable streamwise vortices that enhance near-wall mixing and extend the cooling film’s effectiveness. Axial placement strongly influences vortex–jet interactions: upstream positions allow premature vortex dissipation, while positions too close to the jet disrupt the core flow and induce instabilities. Our study shows that the best cooling occurs for MVGs placed 5–7.5 mm upstream, where vortices remain coherent, jet lift-off is suppressed, and lateral spreading is promoted. Tip angle variations within the tested range have minimal impact. Compared to the baseline case, the best MVG configuration improves surface cooling by 33–46% along the blade, with maximum gains near the root and mid-chord (X/D ≈ 0.05–0.2), highlighting the importance of well-designed MVGs for sustaining effective film cooling across critical blade regions.

## Introduction

Improving the thermal efficiency and power output of modern gas turbines primarily depends on increasing the turbine inlet temperature. These temperatures now often exceed the melting point of the superalloys used in turbine blades, making effective cooling necessary to maintain structural integrity and ensure reliable long-term operation^1,2^. To handle these conditions, turbine blades rely on a combination of internal and external cooling techniques. Among these, film cooling—which introduces a thin layer of coolant over the surface to reduce heat transfer from the hot mainstream flow—remains a widely applied and effective method. Film cooling has been a key topic in turbine thermal management for decades. Reviews by Goldstein ^3^, Bogard et al.^4^, and Han et al.^5^ have shown that its effectiveness depends on several factors, including coolant-hole geometry, inclination angle, blowing ratio, mainstream turbulence, and blade surface curvature. Efforts to improve film cooling generally focus on two aspects: optimizing hole geometry and using external flow-control devices.

A significant industrial development in hole design was the introduction of shaped holes with diffuser-like exits. Bunker^6^ demonstrated that these holes improve coolant attachment and lateral spreading compared to cylindrical holes, and that their performance—by reducing injection momentum and increasing lateral coolant coverage at the exit—is less sensitive to flow variations ^7^. attributed this to the formation of anti-kidney vortex pairs, which also encouraged the development of designs such as sharp-edged diffuser holes^8^, leaf-shaped holes^9^, and trench holes^10^. Multi-jet concepts have been explored to reduce the counter-rotating vortex pair (CRVP) typically observed in single-hole injections. Heidmann and Ekkad^11^ proposed the “anti-vortex” hole with side holes, while Ely and Jubran^12^ numerically showed that sister holes reduce CRVP and improve cooling performance. Wu et al.^13^ confirmed these findings experimentally, and later developments such as Double-Jet Film Cooling (DJFC) by Kusterer et al.^14^ and the “Nekomimi” configuration by Kusterer et al.^15^ expanded these concepts. However, operational conditions strongly influence performance. For instance, Oliver et al.^16^ reported that at high Mach numbers, jet separation and large-scale oscillations can reduce cooling effectiveness by nearly 50%. To optimize shaped hole performance, systematic approaches such as the Box-Behnken method^17,18^ and multi-fidelity models^19^ have been applied. Further studies have examined specific geometric features; for example, laidback fan-shaped holes respond differently to crossflow turbulence depending on whether their corners are sharp or rounded^20^.

Recent work has focused on modifying the external flow near the coolant holes to improve coolant adherence. Devices such as ramps, bumps, and vortex generators (VGs) can influence boundary-layer structures and counteract CRVP effects. Na and Shin^21^ showed that upstream step-ramps suppress horseshoe vortices and increase lateral coolant coverage, while Sakai^22^ found that downstream bumps generate longitudinal vortices that improve film attachment. Funazaki et al.^23^ placed a pair of base-type double-flow-control devices (DFCDs) ahead of the cooling hole on a flat plate and reported improved film cooling effectiveness. More recently, Kawabata et al.^24^ extended this approach to turbine blades and observed comparable improvements in surface cooling performance. In addition to passive techniques, several active flow-control approaches have been proposed to further enhance film cooling. Jet pulsation^25^ and plasma actuation^26^ have shown the potential to improve coolant attachment and coverage by modifying near-wall turbulence structures and reducing jet lift-off. Recent studies have specifically investigated plasma aerodynamic actuation (PAA) combined with pulsed coolant injection. Shen et al.^25^ used large eddy simulations and found that pulsed injection alone increased jet penetration but reduced cooling efficiency by about 15%, whereas adding plasma actuation mitigated lift-off effects from the counter-rotating vortex pair (CRVP) and improved overall efficiency by about 42%. The plasma-induced electrohydrodynamic force weakened coherent vortices, limited excessive mixing, and stabilized the near-wall coolant layer, showing that PAA can actively control vortical evolution and complement traditional methods such as vortex generators. VGs, initially used for boundary-layer control by Babinsky et al.^27^, were later applied to film cooling by Rigby and Heidmann^28^, who numerically showed that downstream VGs can produce downwash vortex pairs. Experiments and LES studies by Zaman et al.^28^ and Shinn and Vanka^29^ confirmed that VGs generate near-wall counter-rotating vortices that help distribute coolant along the surface. Additionally, trenched holes have been studied to increase lateral coolant coverage^31^, and triangular craters have been shown to enhance overall cooling effectiveness^30^. Moreover, in experimental studies, Savari et al.^31^ introduced a novel single-step technique to fabricate high-resolution microscale patterns on both rigid and flexible substrates using DC sputtering with a shadow mask. This approach is particularly well-suited for producing MVGs for film cooling on a stator.

Vortex generators (VGs) provide a highly efficient approach for improving heat transfer and coolant coverage. Their small size and straightforward installation make them attractive for both internal and film cooling applications. Longitudinal vortices generated by VGs are generally more effective than transverse ones in enhancing heat transfer^32^. In film cooling, VGs aim to counteract the adverse effects of the counter-rotating vortex pair (CRVP). For example, the use of a micro ramp in a single-row cooling model demonstrated that the anti-CRVP transports coolant closer to the wall, improving local cooling performance^29^. Numerical studies of VGs, including configurations with rectangular plates downstream of cooling holes, confirm that lateral spreading of the coolant jet is increased^33^. Experimental investigations have shown that the strength of the anti-CRVP grows with both VG height and blowing ratio^34^, and further analysis revealed that the introduced vortices widen coolant coverage while mitigating the magnitude of existing CRVPs^35^. Additionally, VGs have been applied in internal cooling to promote fluid mixing, resulting in significant heat transfer enhancement^36–39^. Multi-row film cooling introduces additional complexity due to interactions between upstream and downstream jets. In double-row arrangements, the upstream ejected coolant can influence downstream effectiveness, sometimes reducing coolant coverage due to the thickened upstream boundary layer^40^. Conversely, studies using the hybrid thermal lattice Boltzmann method indicate that increasing row spacing can widen coolant coverage^41^. Compound angle arrangements have also been explored, showing that in-line configurations may outperform staggered layouts under certain conditions^42^. Overall, both experimental and numerical multi-row studies continue to clarify the interplay between turbulence intensity, blowing ratios, compound angles, and resulting cooling effectiveness^43–45^.

 Although geometric optimization and external flow-control methods have demonstrated some benefits, research on micro vortex generators (MVGs) remains limited. In addition, the variables that influence MVG geometry and their effects on cooling performance have only been examined to a limited extent. Most previous studies focused on flat plates rather than realistic blade surfaces. In this work, several MVG geometries are examined upstream of a coolant injection slot on a stator blade. The study evaluates how variations in MVG height, slope, and installation location affect near-wall flow structures, coolant coverage, and overall film cooling performance, including turbulence kinetic energy (TKE), surface pressure distribution, temperature, film cooling effectiveness, enthalpy, stagnation density, cfRe (friction Reynolds number), and Nusselt number. This research aims to assess the influence of MVG geometry on film cooling effectiveness and to examine how MVGs modify the local flow field and reduce CRVP. Adjusting these parameters can meaningfully influence cooling performance by changing vortex strength, turbulence, and flow separation patterns. In this study, in addition to the initial MVG height of 2.5 mm, heights of 1.5, 4, and 5.5 mm were also considered. For the tip angles, in addition to the initial design of 27°, angles of 32° and 22° were tested. Angles 5° above or below this range were also examined, but since they showed no significant effect on cooling performance, they were not included in this study. Regarding the MVG location, in addition to the initial position of 10 mm upstream of the cooling injection point, positions 5 and 7.5 mm closer and 5 and 7.5 mm further were tested, resulting in five positions: 2.5, 7.5, 10, 12.5, and 17.5 mm. Shorter distances were impractical, and longer distances caused premature flow separation. The ranges of these parameters were selected based on previous studies examining MVG geometries for surface cooling. This setup is intended to evaluate how variations in MVG height, tip angle, and streamwise position influence near-wall flow, vortex formation, and coolant distribution, and to identify configurations that improve film cooling performance and surface protection without reducing overall effectiveness.

## Numerical modeling

**Fig. 1:**
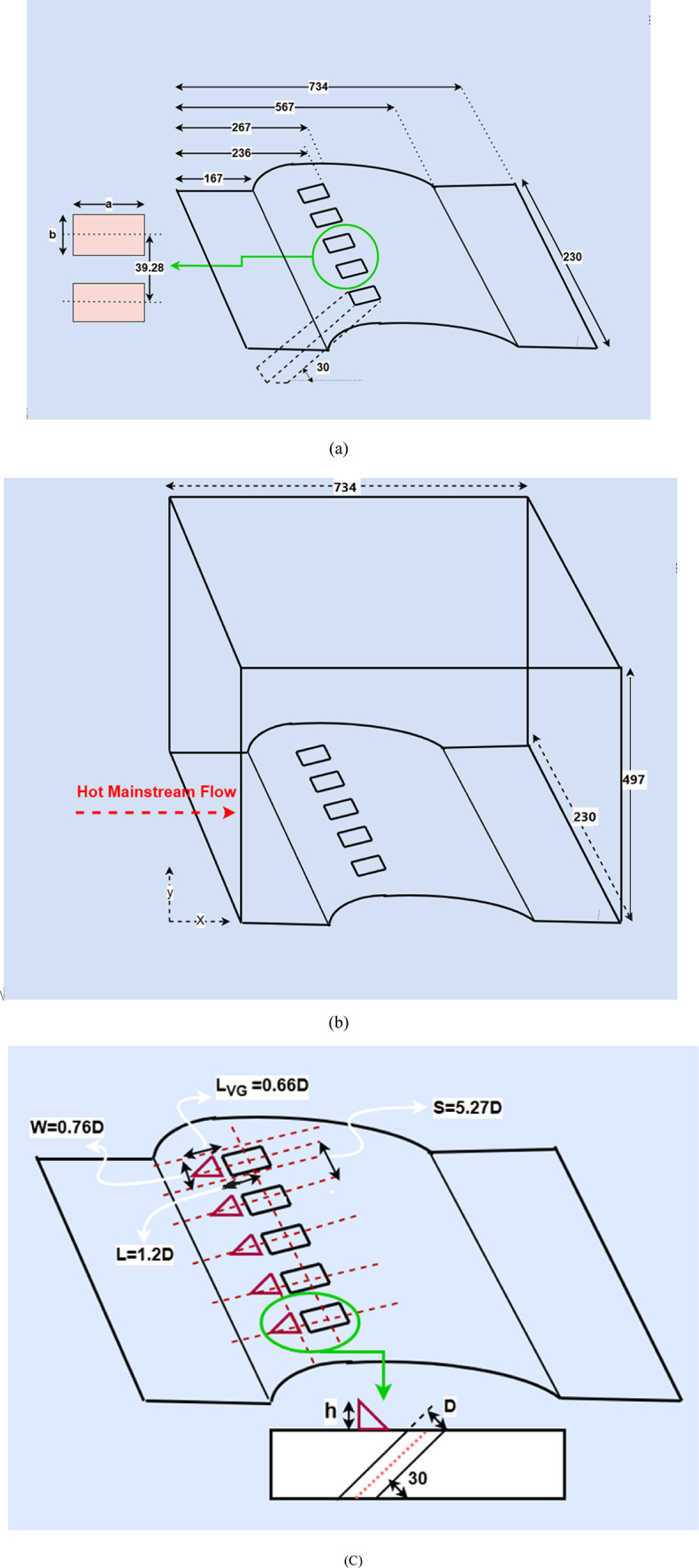
(**a**) Injection openings on a curved surface; (**b**) schematic of the wind tunnel model; (**c**) design and geometry of film cooling slots with a triangular MVG^46,47^.

The sizes and geometry of the cooling holes were adopted from Koça et al.^46^. while the geometry and dimensions of the micro vortex generators were taken from Zheng et al.^47^. The computational domain was based on these validated studies, extending sufficiently upstream, downstream, and laterally to allow full development of the main flow and boundary layer before interacting with the MVGs and cooling holes, and to avoid boundary effects. In this study, we investigate the effects of varying MVG height, tip angle of the triangular pyramid, and streamwise position relative to the coolant injection point on near-wall flow, vortex development, and the interaction between the injected coolant jet and the hot mainstream. These geometric and positional changes alter local mixing, the penetration of coolant into the boundary layer, and convective heat transfer along the blade surface, providing practical insights for optimizing MVG design and improving film cooling performance on realistic turbine blades. For the film cooling investigations, five identical injection holes were drilled in a straight line on a curved surface, each oriented at a 30-degree angle to the surface. All holes are rectangular and have the same cross-sectional area. A single representative geometry is shown in Table 1. Figure 1a illustrates the representative model of these injection holes and their dimensions on the curved surface used in this study. Figure 1b provides a schematic representation of the wind tunnel model. The symbol c denotes the blade thickness. The geometry and dimensions of the MVGs upstream of the slots are shown in Fig. 1c.

**Table 1:** Surfaces and injection hole dimensions^46^.

Surface	Section	a (mm)	b (mm)	Area (mm^2^)	c (mm)
A	a b	10	5.68	56.80	6

The numerical setup for film cooling with upstream vortex generators (VGs) is shown in Fig. 1. The model includes a row of five rectangular holes, each with a hydrodynamic equivalent diameter of *D* = 7.24 *mm*. Coolant gas is injected at an angle of *β* = 30° over a curved blade surface. The holes are spaced apart spanwise by *S* = 5.27*D*, which matches typical designs used in gas turbine engines^4^. In the simulation, MVGs are positioned upstream of each cooling hole, with their axial centers aligned with the holes’ centers. The length and width of each MVG are set to ${L}_{VG}=0.66D$ and $W=0.76D$, respectively. The distance between the MVG’s trailing edge and the center of the cooling hole is denoted as $L=1.2D$. As a result, the initial placement of the MVGs was set 3.94 mm upstream of the injection hole’s leading edge.

Using ANSYS Meshing, the flat plate, curved surfaces, and wind tunnel were modeled in three dimensions and analyzed with FLUENT CFD. Table 2 summarizes the solver setup and key numerical parameters, including the turbulence model, near-wall treatment, discretization schemes, convergence criteria, under-relaxation factors, and mesh details, ensuring accurate and stable results.

**Table 2:** Solver setup and key numerical parameters.

Parameter	Setting/value
Solver type	Pressure-based, implicit, segregated (steady-state)
Pressure–velocity coupling	SIMPLE algorithm (flux type: Rhie-Chow)
Spatial discretization	Gradient: least squares cell-based Pressure: PRESTO! Momentum: QUICK Energy: QUICK Turbulent kinetic energy (*k*): QUICK Turbulent dissipation rate (*ε*): QUICK
Pseudo time method	Off (steady-state)
Turbulence model	Standard *k–ε* with two-layer wall treatment
Near-wall treatment	Inflation layers: number of layers = 4; growth rate = 1.1; smooth transition; transition ratio = 0.272; inflation algorithm = pre; target y⁺ ≈ 30–300
Residual convergence	Energy ≤ 1 × 10⁻⁶; others ≤ 1 × 10⁻^5^
Monitor convergence	Film cooling effectiveness *η* variation < 1 × 10⁻^4^
Under-relaxation factors	Pressure: 0.3–0.5; momentum: 0.7; *k, ε*: 0.8
Mesh	Hybrid: hexahedral in mainstream, tetrahedral near MVG/holes; total cells = 192,352
Fluid properties	Ideal gas; constant except *ρ(T)* via the ideal gas law
Non-modeled physics	Radiation and solid conduction are neglected (adiabatic walls)

Periodic boundary conditions were applied in the simulation due to the repetitive nature of the computational domain and the symmetric design of the jet injection cavities. A single cavity with a vortex generator positioned upstream of the flow was used instead of modeling multiple grooves to simplify the model and reduce computational costs. All subsequent analyses were based on this simplified configuration. The boundary conditions applied in the current simulation are summarized in Table 3.

**Table 3:** Boundary conditions used in the present numerical simulation.

Boundary/condition	Type/description	Value/setting in FLUENT
Mainstream inlet	Velocity inlet	*V* = 15 m/s; *T* = 300 K; turbulence intensity = 1%; turbulent viscosity ratio = 10
Coolant inlet (film holes)	Velocity inlet	*Vj* = 6.59 m/s (*M* = 0.54); *Tj* = 600 K; turbulence intensity = 1%; turbulent viscosity ratio = 10
Outlet	Pressure outlet	Static pressure = 101,325 Pa; backflow *T* = 300 K
Blade/MVG/tunnel walls	Wall (No-slip)	Adiabatic; no-slip condition; two-layer wall treatment
Lateral sides	Periodic/symmetry	Periodic boundary conditions for repeated cavities (spanwise periodicity)
Fluid model	Material/state	Ideal gas; constant properties except *ρ(T)* via ideal gas law
Turbulence model	Turbulence closure	Standard *k–ε* with two-layer wall treatment
Near-wall/boundary layer (Inflation)	Inflation layers	Number of layers = 4; growth rate = 1.1; smooth transition; transition ratio = 0.272; inflation algorithm = pre; target y+ ≈ 30–300
Wall treatment/y+ target	Near-wall resolution	Target *y* + = 30–300; log-layer region (valid for wall-function approach)
Solver type	Pressure–velocity algorithm	Pressure-based, implicit, segregated solver (steady-state); SIMPLE algorithm
Discretization schemes	Spatial schemes	Momentum, energy, turbulence: second-order upwind; pressure: PRESTO!
Convergence criteria	Residuals/monitors	Residuals ≤ 1e − 6 for energy, 1e − 5 for others; *η* variation < 1e − 4
Under-relaxation	Numerical stability	Typical: pressure 0.3–0.5, momentum 0.7, *k, ε* = 0.8
Mesh	Type/refinement	Hybrid: hexahedral in mainstream, tetrahedral near MVG/holes; total cells = 192,352
Physical non-models	Radiation/conduction	Radiation and solid conduction are neglected (adiabatic walls)
Characteristic parameters	Flow/geometry	*D* = 7.24 mm; *Re_D* ≈ 27,400; hydraulic diameter = 314.4 mm; *M* = 0.54

**Fig. 2:**
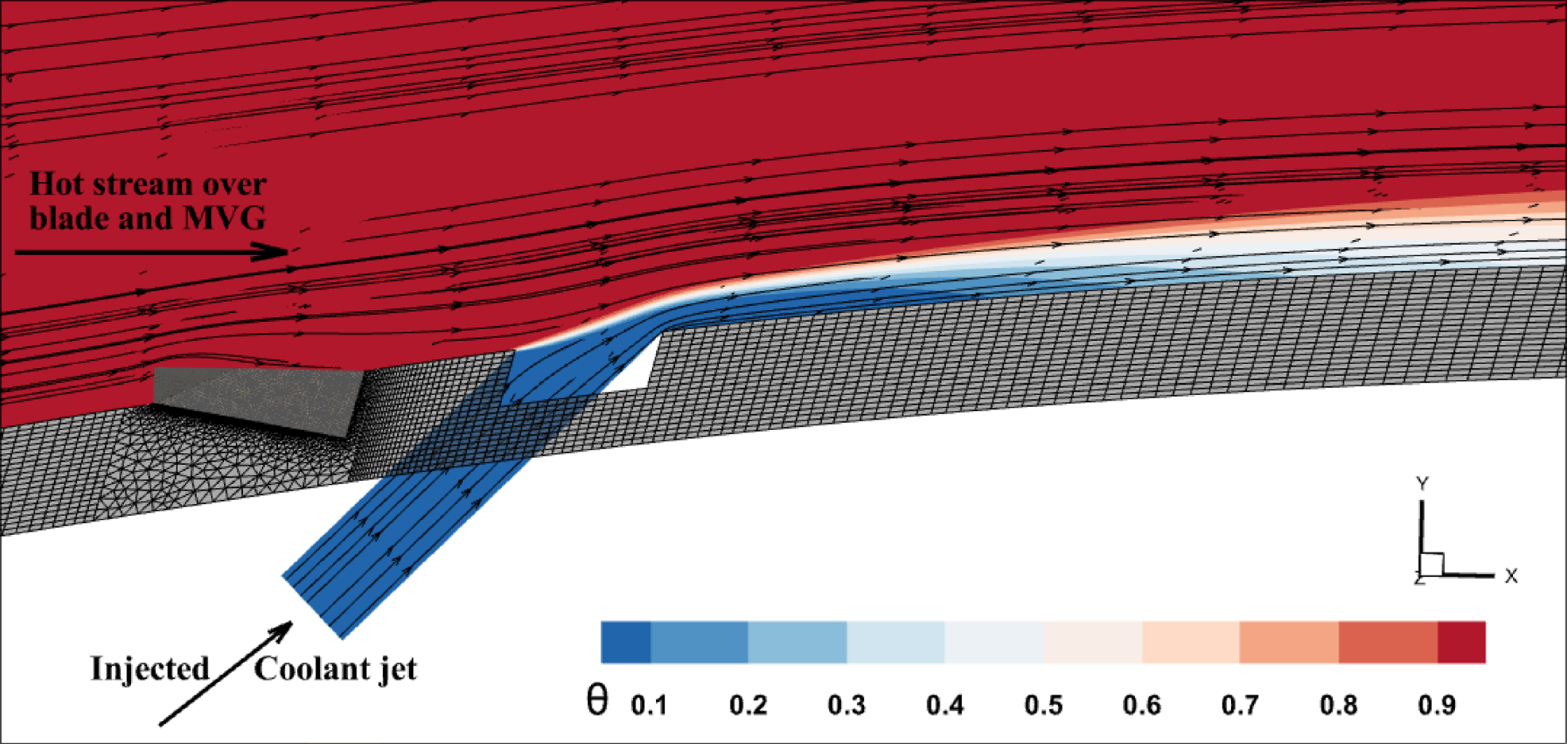
Film cooling through a jet injection with upstream MVG.

**Fig. 3:**
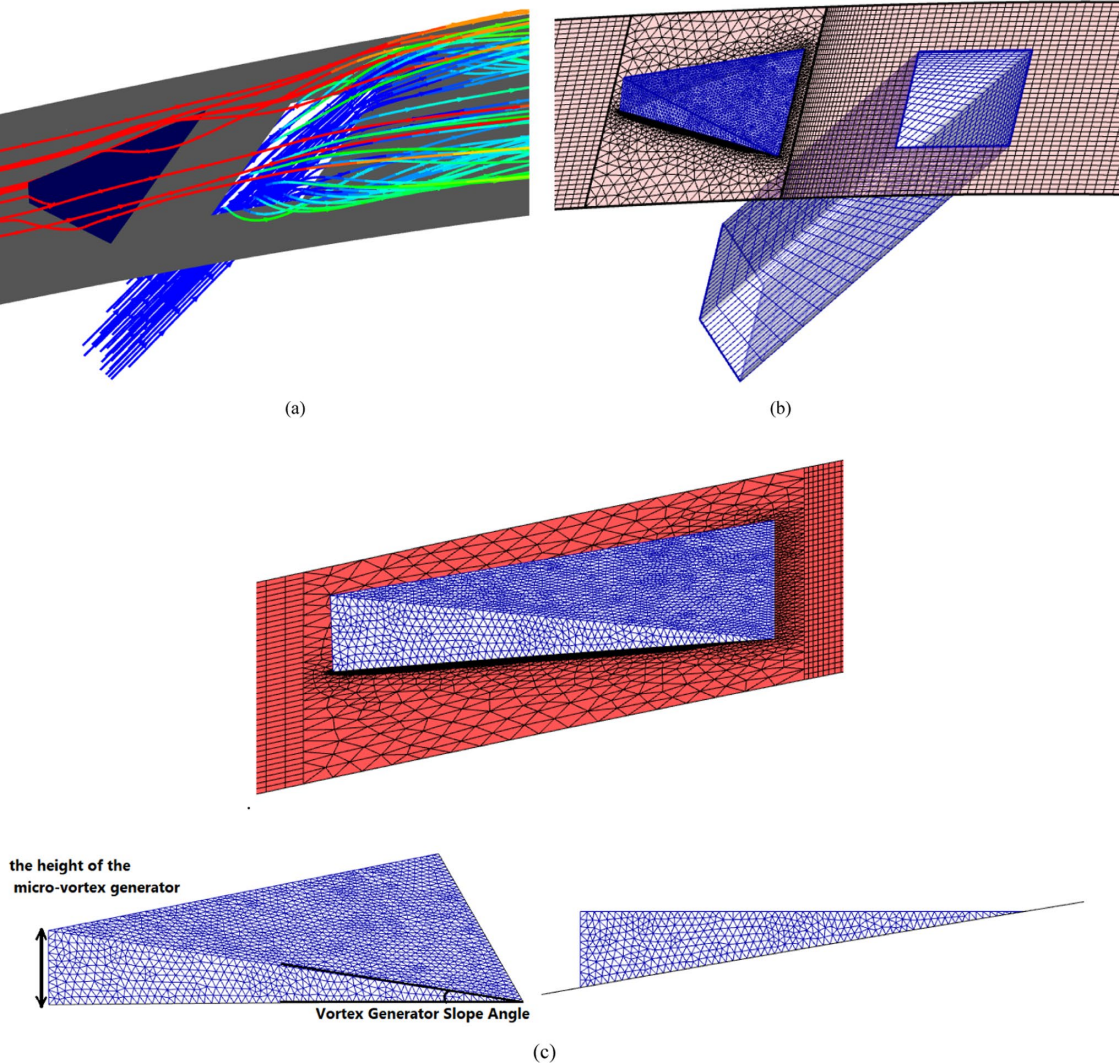
(**a**) Streamlines of the injected cooling jet interacting with the hot flow over the blade; (**b**) mesh layout showing hexahedral elements for the tunnel and tetrahedral elements for the hole and MVG; (**c**) MVG: mesh, height, and inclination angle.

Figures 2 and 3 show the effect of the MVG on the cooling performance of the stator blade surface. The hot airflow passes from left to right across the blade, and symmetric boundary conditions were applied to both sides. Figure 2 shows the temperature field in dimensionless form (θ), based on a normalization where the minimum and maximum temperatures correspond to a ratio of 1:2. In actual values, this range corresponds to 300–600 K. This dimensionless representation of temperature is applied consistently across all figures and analyses in the manuscript.

Figure 2a, illustrates the streamlines of the injected cooling jet interacting with the unsteady hot flow over the blade, highlighting the complex flow behavior that results from this interaction. Figure 3b, also presents the mesh layout for the wind tunnel, injection holes, and MVG. Hexahedral elements were used for the main tunnel structure, while tetrahedral elements were applied to the holes and the MVG to better capture their geometry. The mesh is significantly refined around the injection holes to ensure accurate resolution of local flow features. Figure 3c focuses on the MVG’s mesh characteristics, including its height and inclination angle. The baseline MVG used in this study has a tip height of 2.5 mm, an inclination angle of 27°, and is positioned 10 mm upstream of the cooling hole.

**Fig. 4:**
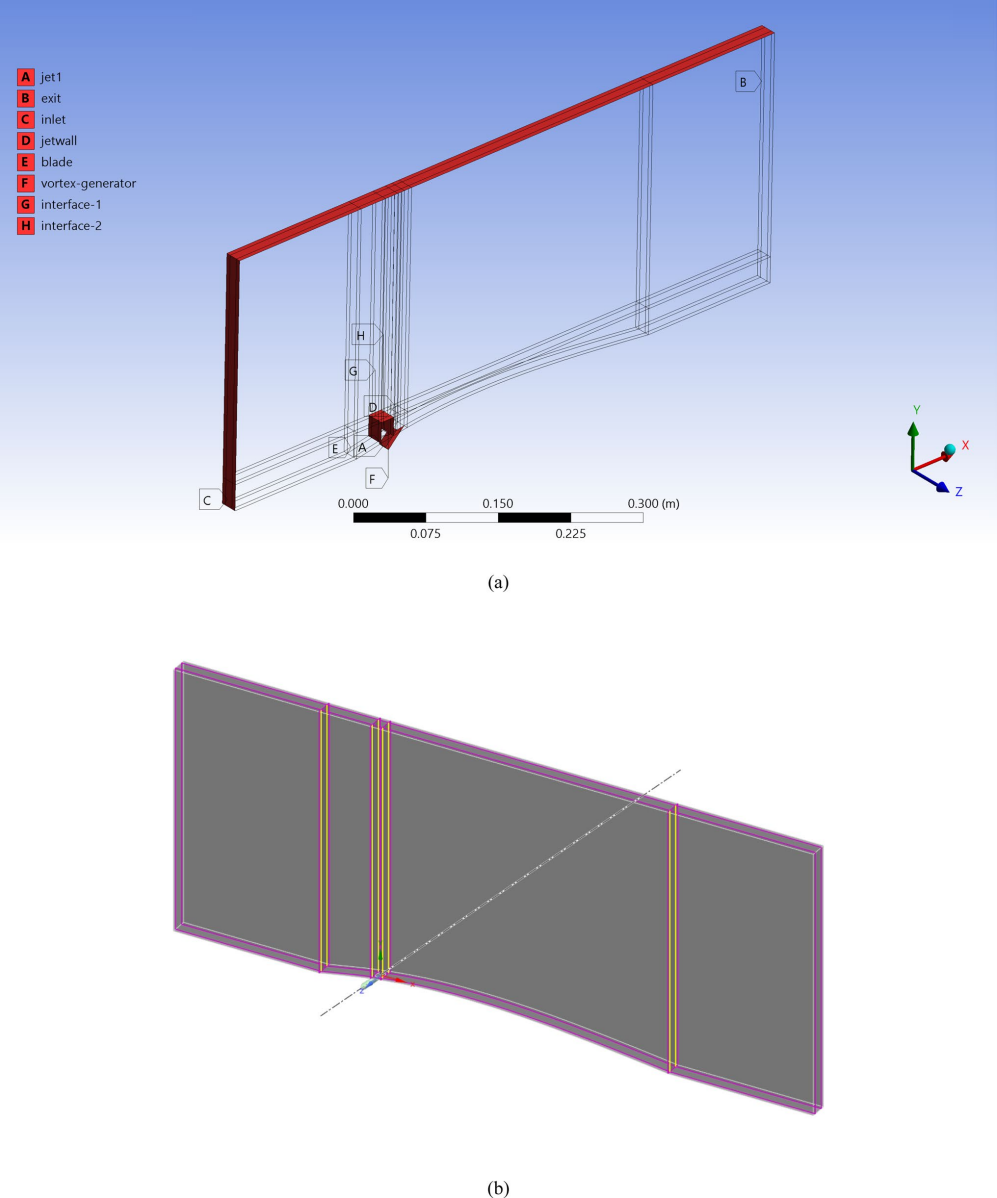

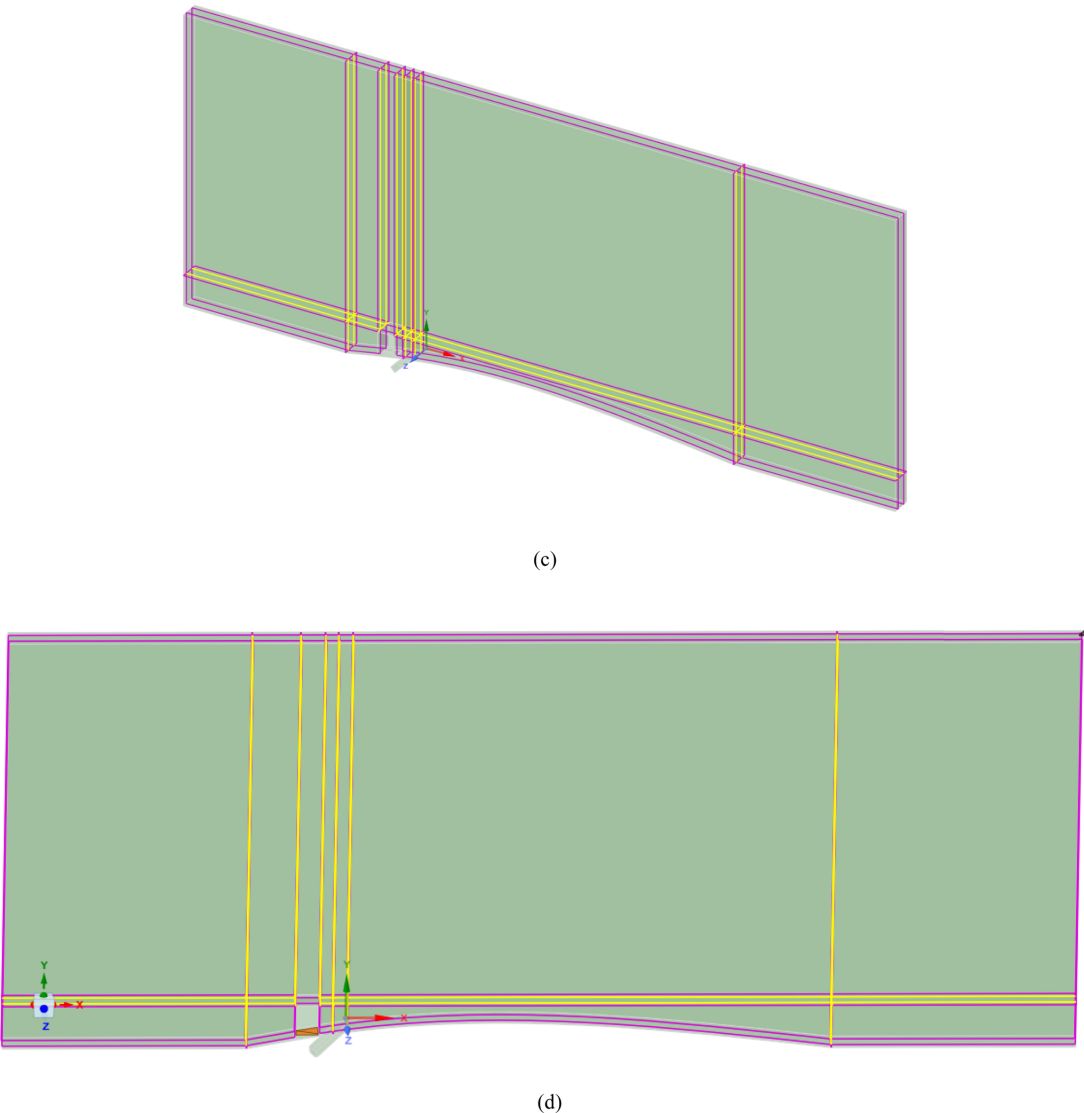

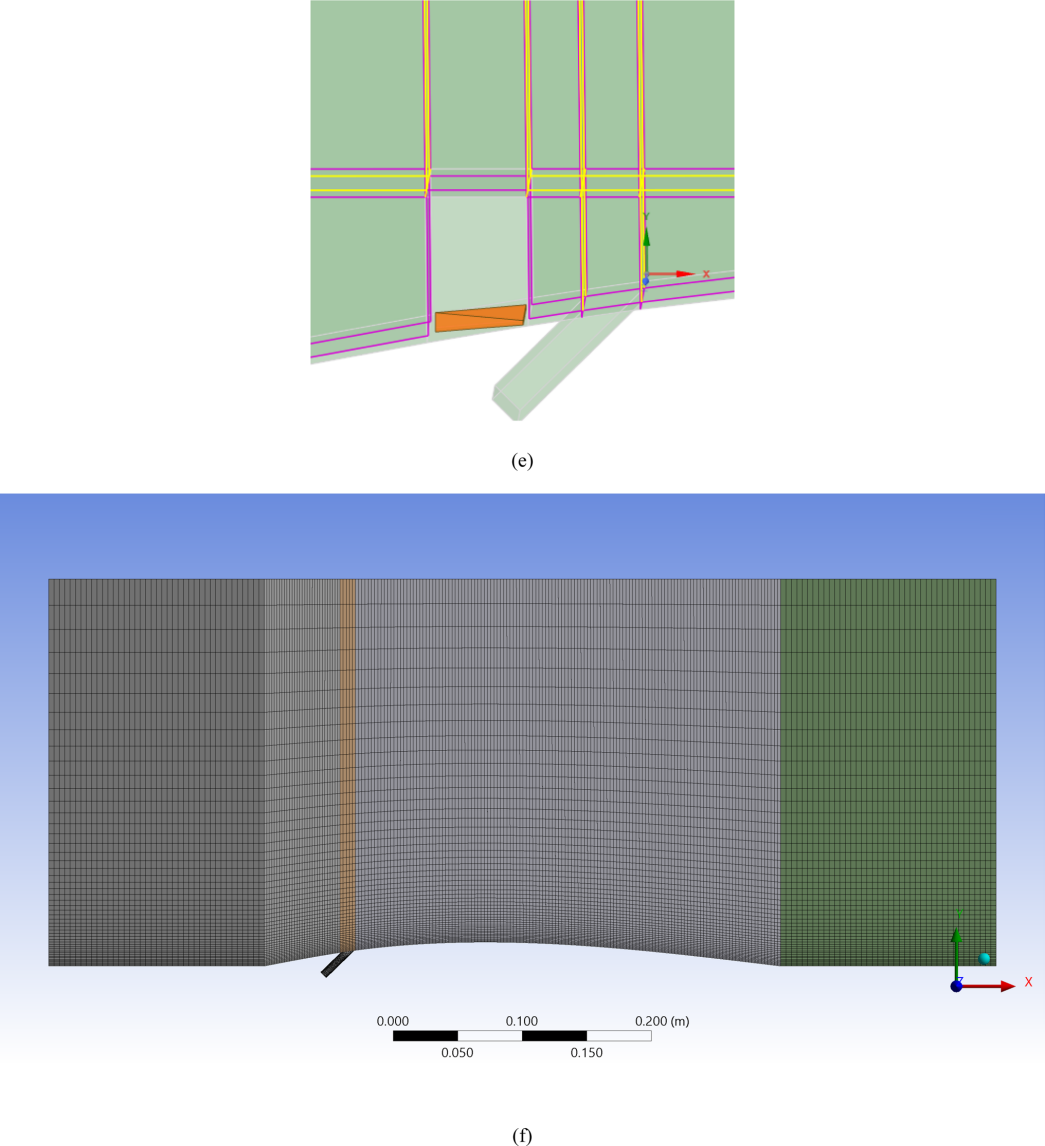

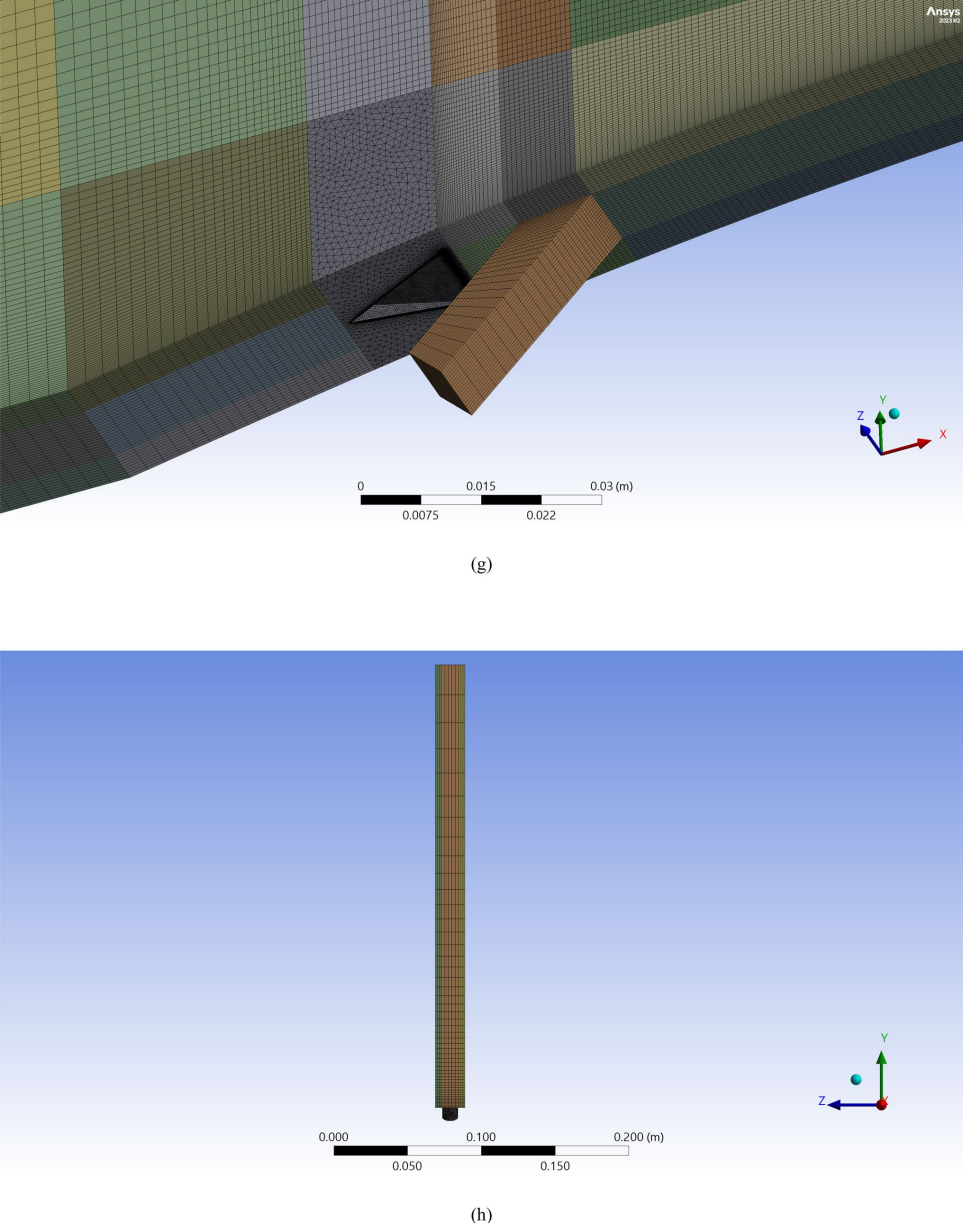

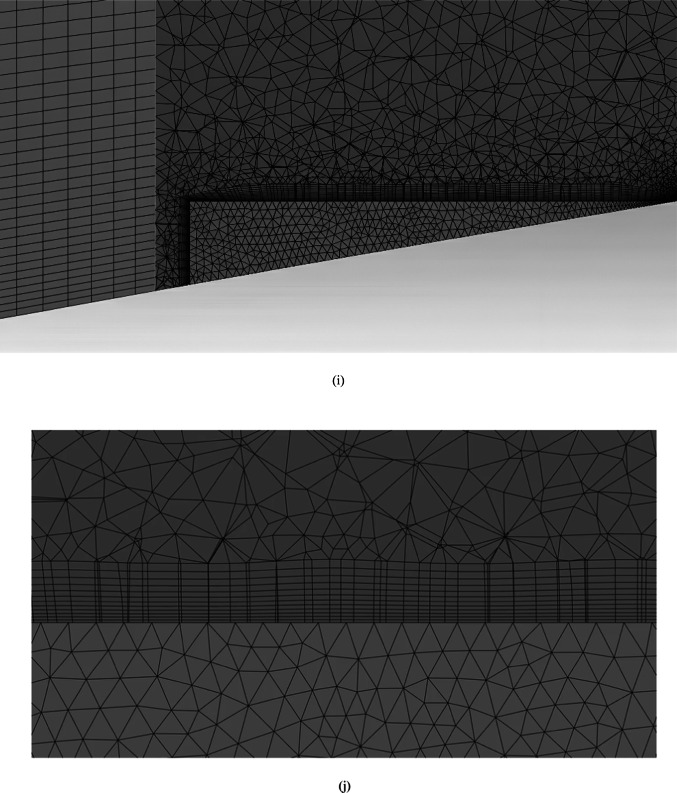
(**a**) Computational domain and boundary conditions for the blade with MVG; (**b**) coolant injection hole location; (**c**, **d**) block-structured mesh configuration around the MVG and within the computational domain; (**e**) close-up view of the MVG installation region; (**f**) surface mesh of the blade and computational domain at the plane *z* = 0; (**g**) detailed mesh arrangement around the MVG; (**h**) cross-sectional mesh of the blade; (**i**) boundary-layer grid refinement around the MVG region.; (**j**) refined near-wall mesh over the blade surface.

Figure 4a shows the CFD domain and boundary conditions for the blade with the MVG, and Fig. 4b indicates the coolant jet injection hole location. A triangular MVG with a positive slope is positioned upstream of the flow, just behind the injection area. A dedicated block mesh was generated around the MVG to enhance grid accuracy. Hexahedral elements were applied for the blade and slot, while a tetrahedral mesh was employed to capture the MVG’s complex geometry. Figure 4c, d illustrate the block-structured mesh around the MVG and the full computational domain. Figure 4e provides a close-up view of the MVG region; Fig. 4f presents the overall blade mesh at z = 0; Fig. 4g shows the detailed mesh around the MVG; and Fig. 4h displays the blade’s cross-sectional mesh. Additionally, Fig. 4i shows the boundary-layer grid refinement around the MVG region, while Fig. 4j shows the refined near-wall mesh on the blade surface.

This study relies on two primary sets of equations: the governing equations for fluid flow and the heat conduction equation for the solid region^48^. For the fluid domain, we solve the steady-state, three-dimensional Navier–Stokes equations, which encompass the conservation equations for mass, momentum, and energy, as shown in Eqs. (1)–(3).

1 $$\frac{\partial }{{\partial x_{i} }}\left( {\rho u_{i} } \right) = 0$$

2 $$\frac{\partial }{{\partial x_{i} }}\left( {\rho u_{i} u_{j} } \right) = \rho \vec{g}_{j} - \frac{\partial P}{{\partial x_{j} }} + \frac{\partial }{{\partial x_{i} }}\left( {\tau_{ij} - \rho \overline{{u_{i}{\prime} u_{j}{\prime} }} } \right)$$

3 $$\frac{\partial }{{\partial x_{i} }}\left( {\rho c_{p} u_{i} T} \right) = \frac{\partial }{{\partial x_{i} }}\left( {\lambda \frac{\partial T}{{\partial x_{i} }} - \rho c_{p} \overline{{u_{i}{\prime} T^{\prime}}} } \right) + \mu \phi$$

In these equations, the symmetric stress tensor is defined as shown in Eq. (4).

4 $$\tau_{ij} = \mu \left( {\frac{{\partial u_{j} }}{{\partial x_{i} }} + \frac{{\partial u_{i} }}{{\partial x_{j} }} - \frac{2}{3}\delta_{ij} \frac{{\partial u_{k} }}{{\partial x_{k} }}} \right)$$

In these equations, μϕ represents the dissipation due to turbulence and $\lambda$ denotes the thermal conductivity. The terms $\rho \overline{{u_{i}{\prime} u_{j}{\prime} }}$ and $\rho c_{p} \overline{{u_{i}{\prime} T^{\prime}}}$ represent the Reynolds stresses and heat fluxes in turbulent flow, respectively. These terms are zero for laminar flows but are accounted for in turbulence models. Various turbulence models are available for analyzing thermal and fluid dynamic problems. One of the commonly used models is the *k-ε* model, which is based on the Boussinesq hypothesis^49^. In this model, the Reynolds stresses are expressed using the relationship given in Eq. (5).

5 $$- \rho \overline{{u_{i}{\prime} u_{j}{\prime} }} = \mu_{t} \left( {\frac{{\partial u_{i} }}{{\partial x_{j} }} + \frac{{\partial u_{j} }}{{\partial x_{i} }}} \right) - \frac{2}{3}\rho k\delta_{ij}$$

In this context, $k$ signifies turbulent kinetic energy, while $\mu_{t}$ represents turbulent viscosity. The latter can be derived from Eq. (6), which is essential for modeling turbulence effects in fluid flow.

6 $$\mu_{t} = \frac{{\rho C_{\mu } k^{2} }}{\varepsilon }$$

In Eq. (6), $\mu_{t}$ is a constant, and $\varepsilon$ represents the dissipation rate. The relationships for turbulent kinetic energy (k) and the dissipation rate (ε) are presented in Eqs. (7) and (8).

7 $$\frac{\partial }{{\partial x_{i} }}\left( {\rho u_{i} k} \right) = \frac{\partial }{{\partial x_{i} }}\left[ {\left( {\mu + \frac{{\mu_{t} }}{{\sigma_{k} }}} \right)\frac{\partial k}{{\partial x_{i} }}} \right] + G_{k} - \rho \varepsilon$$

8 $$\frac{\partial }{{\partial x_{i} }}\left( {\rho u_{i} \varepsilon } \right) = \frac{\partial }{{\partial x_{i} }}\left[ {\left( {\mu + \frac{{\mu_{t} }}{{\sigma_{\varepsilon } }}} \right)\frac{\partial \varepsilon }{{\partial x_{i} }}} \right] + C_{1\varepsilon } G_{k} \frac{\varepsilon }{k} - C_{2\varepsilon } \rho \frac{{\varepsilon^{2} }}{k}$$

The term $G_{k}$ denotes the generation of turbulent kinetic energy resulting from velocity gradients. The turbulent heat flux is represented by the turbulent thermal conductivity $\lambda_{t}$, as outlined in Eq. (9):

9 $$\rho c_{p} \overline{{u_{i}{\prime} T^{\prime}}} = - \lambda_{t} \frac{\partial T}{{\partial x_{i} }} = - c_{p} \frac{{\mu_{t} }}{{{\mathrm{Pr}}_{t} }}\frac{\partial T}{{\partial x_{i} }}$$

The constants used in the equations, such as $C_{1\varepsilon }$, $C_{2\varepsilon }$, $C_{\mu }$, $\sigma_{k}$, and $\sigma_{\varepsilon }$, are specified as follows: $C_{1\varepsilon }$ = 1.44, $C_{2\varepsilon }$ = 1.92, $\sigma_{\varepsilon }$ = 1.3, $\sigma_{k}$ = 1.0, and $C_{\mu }$ = 0.09^50^. Additionally, the Prandtl number is considered to be 0.85. While these values should ideally be defined for the specific flow physics, such as accelerated flows, flows with separation, low Reynolds number flows, curved flows, and rotating flows, they are commonly employed in similar analyses due to their widespread acceptance. Therefore, this study adopts these values for consistency with previous research. The description of the Enhanced Wall Treatment used in conjunction with the standard *k–ε* model is provided below. This model is commonly applied in internal cooling flows with moderate Reynolds numbers, where fully developed turbulence may not be present throughout the entire domain. To improve near-wall resolution, particularly in regions where turbulence is underdeveloped, the two-layer approach is employed. This method divides the near-wall region based on the turbulent Reynolds number $\left( {Re_{y} } \right)$ into a viscosity-affected sublayer and a fully turbulent outer layer. The combination of wall functions and the two-layer model helps maintain accuracy near the wall while keeping the computational cost reasonable. The turbulent Reynolds number is defined by Eq. (10).

10 $${\mathrm{Re}}_{y} = \frac{{yk^{\frac{1}{2}} }}{v}$$

In this context, the turbulent kinetic energy is denoted as $k$, while $y$ represents the distance from the wall. The standard *ε-k* model is employed for Reynolds numbers greater than 200, applicable in the fully turbulent region. In this area, a wall function approach is utilized, referencing the Wolfstein equations for $Re_{y}$ < 200 to define the affected-viscosity region concerning $y$^51^. Additionally, the transitional region’s turbulent viscosity is derived as a combination of the viscosities from both the fully turbulent and affected-viscosity regions, using a mixing parameter θ:

11 $$\mu_{{t,{\text{ enhanced }}}} = \theta \mu_{t} + \left( {1 - \theta } \right)\mu_{t,l}$$

In Eq. (11), $\mu_{t}$ refers to the turbulent viscosity used in the *k-ε* model for Reynolds numbers exceeding 200, while $\mu_{t,l}$ indicates the viscosity near the wall according to the wall function model. The mixing parameter at the wall is set to 0, while in the fully turbulent region, it is defined as 1. To create suitable wall functions in the near-wall area, linear laws for laminar flow are combined with logarithmic laws for turbulent flow. Furthermore, the governing energy equations for the solid region are defined in Eq. (12). In this context, $k$ represents the thermal conductivity, while $T$ denotes the temperature.

12 $$\nabla \cdot \left( {k\nabla T} \right) = 0$$

This study focuses on heat transfer between two fluids, specifically hot air and the cold injected jet, without considering the thermal conductivity of the solid material (the blade). The adiabatic cooling effectiveness is considered an independent metric, as it is primarily influenced by flow parameters and temperature rather than solely by the material’s thermal properties. In other words, this effectiveness depends on flow characteristics and temperature, including inlet temperatures, flow velocity, and boundary conditions. While the material’s thermal properties can affect adiabatic cooling performance, the effects of temperature and flow are more significant. Thus, adiabatic cooling performance is predominantly governed by flow patterns and thermal conditions. For the simulations, the standard *k–ε* turbulence model was employed due to its validated performance in similar film cooling studies and its balance between accuracy and computational cost. To resolve the near-wall region, the two-layer wall treatment approach was applied in combination with standard wall functions, and the energy equation was enabled. Although more advanced models, such as *SST k–ω* and RSM, provide improved near-wall predictions, the standard *k–ε* model offered a robust and practical choice for the present parametric investigation involving MVG configurations^46,52,53^.

In this study, numerical simulations were conducted under steady-state conditions to investigate the performance of film cooling over a curved stator blade equipped with MVGs. Validated setups from the literature defined the boundary conditions and computational framework to ensure both physical realism and reproducibility. At the domain inlet, a velocity-inlet condition was applied to simulate the mainstream hot flow with a constant velocity of 15 m*/s* and a temperature of 300 K. The cooling air was injected through rectangular film cooling holes positioned upstream on the blade surface. These inlets were also modeled using velocity-inlet boundaries, with injection velocities calculated from a blowing ratio of 0.54, yielding a jet velocity of approximately 6.59 m*/s*. The coolant temperature was set to 600 K, establishing a 300 K thermal gradient across the film layer. At both the main inlet and the coolant inlets, turbulence intensity was prescribed as 1%, and the turbulence viscosity ratio was fixed at 10 to reflect typical low-turbulence wind tunnel conditions. The outlet of the computational domain was treated as a pressure-outlet boundary, maintained at atmospheric pressure (101,325 Pa). All solid surfaces—including the stator blade, the MVG, and the inner tunnel walls—were assumed adiabatic and subject to no-slip conditions. Heat conduction through the solid body and radiative heat transfer were neglected. Due to the periodic arrangement of cooling holes and flow structures, periodic boundary conditions were imposed on the lateral sides of the domain.

Mesh generation was performed using a hybrid scheme, with structured hexahedral elements in the mainstream and upper flow regions. In contrast, unstructured tetrahedral elements were employed near the complex geometry of the cooling holes and MVGs. Local mesh refinement was applied in regions of high velocity and temperature gradients, especially around the hole exits and vortex structures. The mesh near the walls was designed to ensure that wall-adjacent cells remained within the logarithmic layer of the boundary layer, thereby maintaining the validity of the wall-function approach. The working fluid was treated as an ideal gas, and all flow properties were assumed constant except density, which varied with temperature according to the ideal gas law.

The effectiveness of film cooling was evaluated using the adiabatic film cooling effectiveness formulation, which was computed along the streamwise centerline downstream of the cooling holes. To validate the numerical model, results for the baseline case without MVG were compared with experimental data from^46^, showing good agreement in both magnitude and trend of the effectiveness profile. This agreement confirms the suitability of the numerical approach and the applied boundary conditions for predicting film cooling performance in such configurations. The hydraulic diameter of the main airflow was 314.4 mm, and the jet injection orifice diameter was 7.24 mm. The Reynolds number based on the cooling hole diameter and injection conditions was approximately 27,400 at ambient pressure. We have:

13 $${\text{M = }}\frac{{\rho_{j} {\text{ v}}_{j} }}{{\rho_{\infty } {\text{ v}}_{\infty } }}$$

In this context, $M$ represents the blowing ratio, $\rho_{j}$ denotes the density of the injected fluid, $v_{j}$ is the velocity of the injected fluid, $\rho_{\infty }$ is the density of the main flow, and $v_{\infty }$ is the velocity of the main flow. the velocities of the injected fluid $v_{j}$ were calculated using Eq. (13). One factor influencing film cooling effectiveness is the momentum flux ratio, which provides a physical rationale for the selection of blowing ratios. The momentum flux ratio can be calculated using Eq. (14):

14 $${\text{I = }}\frac{{\rho_{j} {\text{ v}}_{j}^{2} }}{{\rho_{\infty } {\text{ v}}_{\infty }^{2} }}$$

For a turbulent boundary layer over a curved blade surface, the skin friction coefficient (*Cf*) is influenced by the local Reynolds number and the surface curvature. Considering the convex curvature of the blade, *Cf* can be expressed using Eq. (15):

15 $$C_{f} = \frac{0.455}{{\left( {\log Re_{x} } \right)^{2.58} }} \times \left( {1 + 0.3\frac{{\updelta }}{R}} \right)$$

where $Re_{x}$ is the local Reynolds number, *R* is the radius of curvature, and δ represents the boundary layer thickness. The presence of curvature alters the flow structure, leading to reduced boundary-layer separation and enhanced turbulent mixing. The Reynolds number, based on the streamwise coordinate *x*, is given by Eq. (16):

16 $$Re_{x} = \frac{Ux}{{\upnu }}$$

, where *U* is the freestream velocity, and *ν* is the kinematic viscosity. This parameter dictates the evolution of the turbulent boundary layer along the blade surface. To evaluate heat transfer performance, the local Nusselt number $\left( {Nu_{x} } \right)$ is defined by Eq. (17).

17 $$Nu_{x} = 0.0296Re_{x}^{0.8} Pr^{\frac{1}{3}} \times \left( {1 + 0.3R_{{\updelta }} } \right)$$

where *Pr* is the Prandtl number. In the numerical analysis, the local Nusselt number was also obtained from the surface heat flux as expressed in Eqs. (18) and (19)^54,55^.

18 $$Nu_{x} = \frac{{h_{x} D}}{{k_{f} }}$$

19 $$h_{x} = \frac{{q_{w}^{{^{\prime\prime}}} }}{{T_{\infty } - T_{w} }}$$

where $q_{w}^{{^{\prime\prime}}}$ is the wall heat flux, $T_{w}$ is the local wall temperature, and $T_{\infty }$ is the mainstream temperature. Since $D$ and $k_{f}$ are always positive, the sign of $Nu_{x}$ depends on $q_{w}^{{^{\prime\prime}}}$. Negative values of $Nu_{x}$ indicate local heat transfer reversal, where the wall transfers heat to the fluid. This occurs in regions affected by recirculation and the impingement of the hot mainstream flow on the MVG surface. The curvature correction factor accounts for the enhanced heat transfer resulting from increased mixing at convex surfaces. Furthermore, the relationship between $C_{f}$ and the friction Reynolds parameter $\left( {cfRe} \right)$ is given by Eq. (20).

20 $$cfRe = C_{f} \times Re_{x}$$

This formulation directly accounts for the influence of both the local Reynolds number and surface curvature on frictional effects. By incorporating curvature corrections, these expressions provide a more accurate representation of flow behavior and heat transfer over the convex blade surface.

**Fig. 5:**
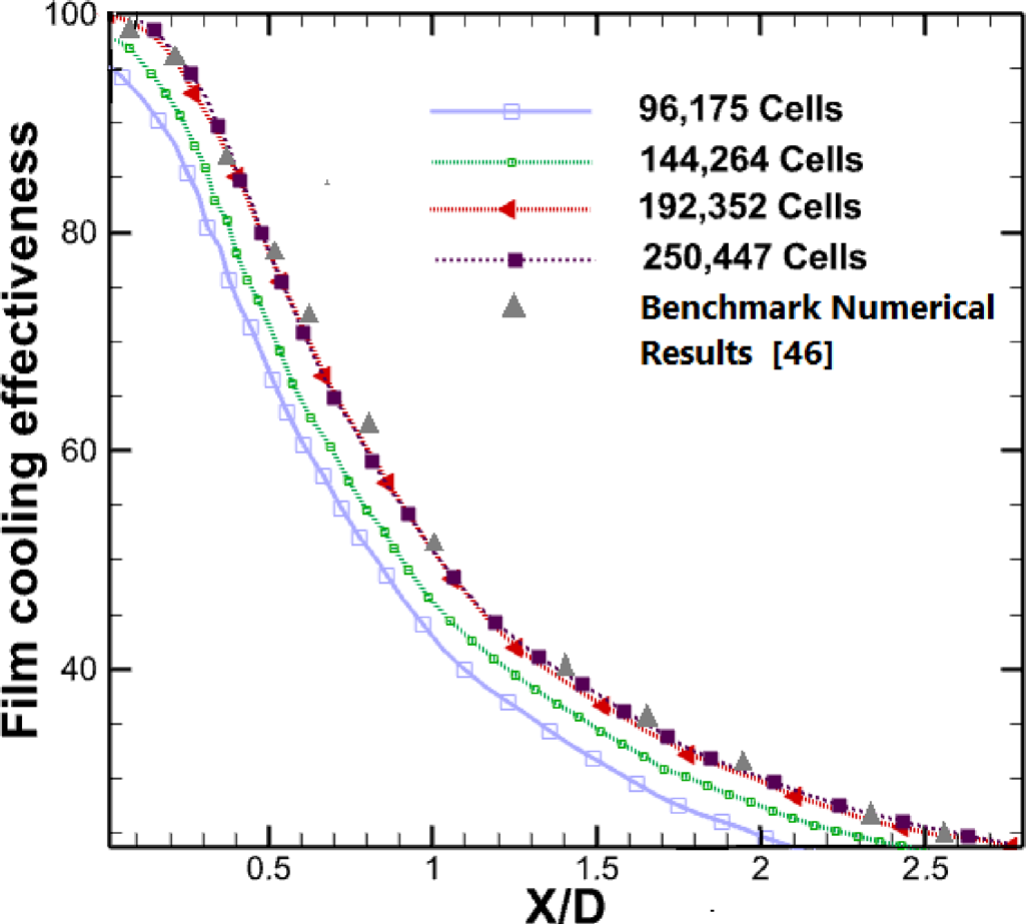
Grid sensitivity analysis: validation of mesh independence through comparison with benchmark numerical results from^46^.

To ensure accurate numerical results without excessive computational demands, the effect of grid size on the results was investigated. The number of grid cells on the measurement surface was increased incrementally until stable solutions were reached. The temperature variations on the measurement surface were monitored at each mesh adjustment, and the optimal grid size was selected for each geometry. As shown in Fig. 5, increasing the grid count from 192,352 to 250,447 cells resulted in minimal temperature changes. This increase in the number of cells did not have a significant impact on the results, but only increased computation time and cost. Therefore, 192,352 cells were chosen. The cooling effectiveness on the y-axis refers to the effectiveness at the centerline along the blade. In the present study, the streamwise coordinate is non-dimensionalized as *X/D*, where *x* is the distance downstream from the center of the cooling hole and *D* is the hole diameter. This normalization facilitates meaningful comparison of flow development and cooling effectiveness across different geometries and operating conditions.

To evaluate the cooling performance, the film cooling effectiveness (η) is used as the primary metric. In addition, the near-wall distributions of total (stagnation) enthalpy $\left( {h_{0} } \right)$ and stagnation density $\left( {\rho_{0} } \right)$ are analyzed to provide complementary insight into local thermal loads and mass fluxes. Total enthalpy, $h_{0}$, represents the sum of the flow’s internal (thermal) energy and kinetic energy. Lower values of $h_{0}$ near the wall indicates that less energy is available for heat transfer to the surface, enhancing film cooling effectiveness. Stagnation density, $\rho_{0}$, is defined as the density the fluid would attain if it were brought isentropically to rest. It reflects the local mass flux and momentum of the coolant jet, influencing its attachment and mixing with the hot mainstream. Together, these parameters help quantify how the coolant jet interacts with the main flow and contributes to surface cooling in high-speed, compressible regimes. The three-dimensional temperature field was calculated along the blade surface, and the adiabatic film cooling effectiveness was determined from these temperatures using Eq. (21).

21 $$\eta = \frac{{T_{adiabatic} - T_{\infty } }}{{T_{j} - T_{\infty } }}$$

In this context, $\eta$ represents the film cooling effectiveness,$T_{adiabatic}$ is the adiabatic wall temperature, $T_{\infty }$ denotes the temperature of the main flow, and $T_{j}$ is the temperature of the injected fluid. The results of the surface-cooling effect along the blade’s longitudinal direction were specifically obtained for the central hole in the row of holes embedded in the blade surface. boundary conditions were defined for the inlet and outlet. The inlet includes the main hot flow. For the blade, a no-slip wall condition was applied.

The employed numerical method utilizes the integral form of the equations and a pressure-based solver. Due to the mesh’s complexity and convergence issues, an implicit formulation was used. The flow was analyzed under steady-state conditions. The segregated solver algorithm was selected, which solves the governing equations separately. To study the temperature differences between the main flow and the cooling jet, the heat transfer equation was solved using a turbulence model. The stator blade surface, as well as the walls of the MVG, were treated as adiabatic with zero gauge pressure. After creating the geometry, meshing, and setting the boundary conditions, the flow was ultimately solved under steady-state conditions.

**Fig. 6:**
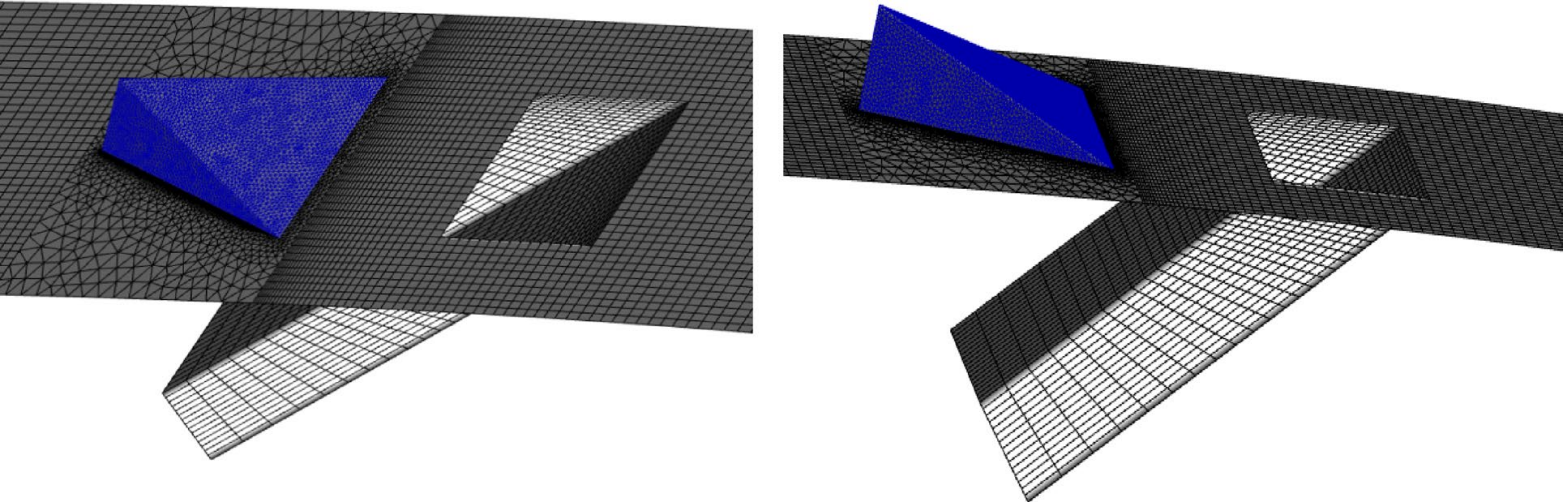
Mesh configuration of the MVG.

Figure 6 displays the MVG mesh configuration, showing its placement upstream of the cooling slot and in front of the freestream hot flow over the blade. Previous research has often focused on the mere presence or absence of MVGs; this work delves into how variations in MVG height, location, and angle relative to the cooling air injection point affect airflow and temperature distribution around the stator blade. By adjusting these parameters, we can significantly improve cooling performance by altering vortex strength, turbulence, and flow separation patterns. This study systematically analyzes three different heights, positions, and angles, providing valuable insights into the aerodynamic mechanisms that enhance convective heat transfer, and laying the groundwork for more effective MVG designs in cooling applications. An optimally designed MVG, positioned to prevent flow separation from the blade surface, enhances mixing between the hot mainstream flow and the cold-injected jet. The goal is to enhance mixing as much as possible so that heat is transferred from the blade surface to the cooling jet, enabling more effective blade cooling.

## Results and discussion

### Impact of vortex generators on film cooling efficiency

**Fig. 7:**
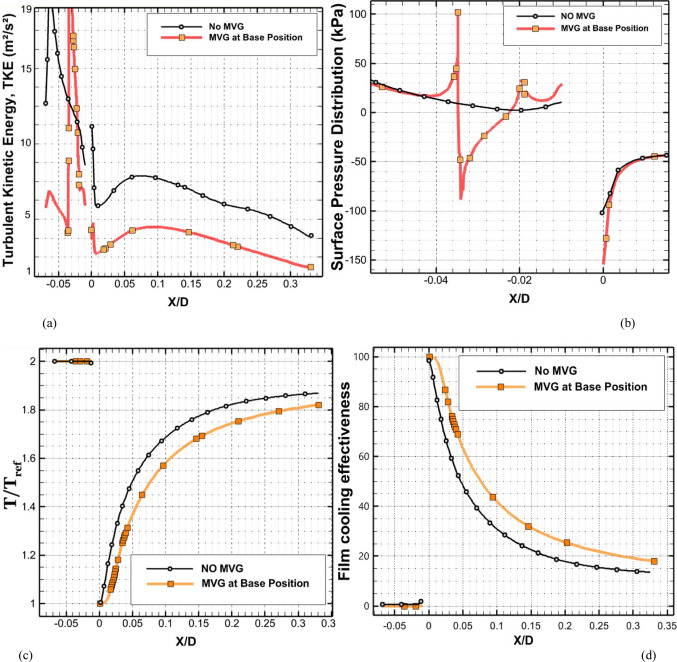

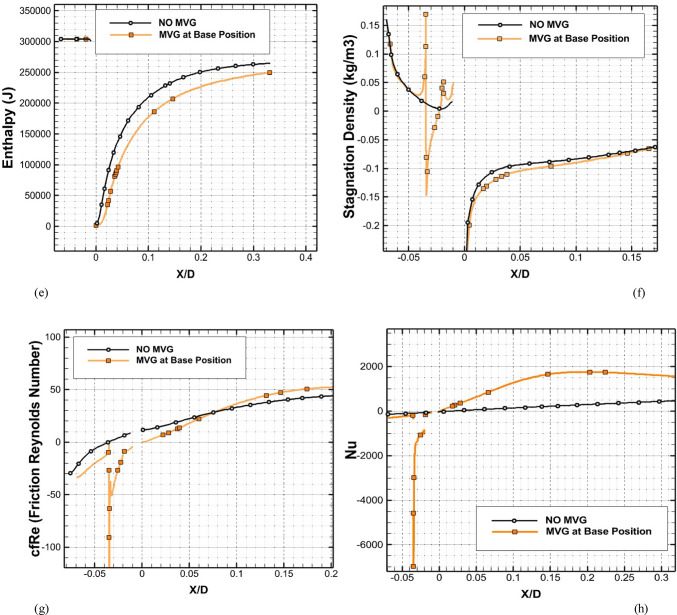
Surface cooling efficiency: a comparison of conditions with and without MVG; (**a**) turbulence kinetic energy; (**b**) surface pressure; (**c**) temperature; and (**d**) film cooling effectiveness; (**e**) enthalpy; (**f**) stagnation density; (**g**) cfRe (Friction Reynolds number); (**h**) Nusselt number.

Figure 7 depicts the behavior of turbulence kinetic energy (TKE), surface pressure distribution, surface temperature, and film cooling effectiveness on the stator blade in two configurations: with and without the baseline triangular MVG. The MVG used in this study has a tip height of 2.5 mm, an inclination angle of 27°, and is installed 10 mm upstream of the cooling hole. The hot mainstream flow approaches from the left side of the blade, first interacts with the MVG, and then reaches the cooling hole, where the coolant jet is injected. When the MVG is installed upstream of the cooling slot, the cooling performance improves significantly, as it promotes better airflow mixing and helps maintain a more uniform cooling layer across the blade surface. For this analysis, variations in temperature and other cooling parameters were examined along a line drawn along the blade’s length and across its mid-width. Data collected along this line were compared for the pre- and post-installation conditions, and corresponding diagrams were generated for both scenarios. Cooling effectiveness in both cases was calculated using Eq. (21).

In Fig. 7a, the distribution of turbulence kinetic energy (TKE) along the normalized distance (*X/D*) is presented for two configurations: one without MVG and another with an MVG placed at the base position. The data clearly illustrate substantial differences in TKE between the two scenarios, underscoring the MVG’s influence on flow dynamics. In the upstream region (*X/D* < 0), the configuration without the MVG shows a high level of turbulence kinetic energy. This indicates the flow is relatively disturbed, likely due to complex flow features or boundary layer separation. In contrast, the MVG configuration exhibits a much lower initial TKE, suggesting that it effectively stabilizes the flow and reduces turbulence at this point. As the flow progresses downstream (*X/D* > 0), the TKE decreases for both configurations. However, the decline is more pronounced in the case without the MVG, indicating rapid dissipation of turbulence energy. The configuration with the MVG, on the other hand, exhibits a more gradual decrease in TKE, suggesting that the micro vortex structures created by the MVG help maintain a level of turbulence that enhances mixing and flow stability. At the injection point (*X/D* = 0), both configurations experience a notable drop in TKE. However, the configuration with the MVG shows a significantly lower TKE than the one without it. This indicates that the MVG stabilizes the flow, allowing for better control over turbulence characteristics. Further downstream, TKE continues to decrease in both cases, but the MVG configuration consistently maintains higher TKE than the no-MVG scenario. This persistent TKE in the MVG configuration is advantageous, as it can improve the mixing of the cooling air with the surrounding hot flow, thereby enhancing cooling effectiveness. Overall, the presence of the MVG significantly alters the distribution of turbulence kinetic energy, reducing initial turbulence and maintaining favorable flow conditions downstream. These findings highlight the significance of MVGs in improving flow characteristics and enhancing cooling performance in high-temperature applications.

As shown in Fig. 7b, the surface pressure distribution (*kPa*) is plotted as a function of the normalized distance (X/D) for two configurations: one without an MVG and the other with an MVG at the base. The data highlight the significant influence of the MVG on surface pressure patterns, which are closely tied to flow behavior and aerodynamic performance. In the upstream region (*X/D* < 0), the configuration without the MVG exhibits a smooth, consistent pressure distribution, indicating undisturbed flow conditions. In contrast, the configuration with the MVG exhibits a sharp drop in pressure, particularly between *X/D* =  − 0.04 and *X/D* =  − 0.02. This significant negative pressure spike indicates the MVG’s impact in disrupting the flow and generating vortex structures. Following this initial drop, the pressure gradually recovers, suggesting that the induced vortices begin to stabilize as they move downstream. At the injection point (*X/D* = 0), both configurations exhibit a rapid rise in surface pressure. However, the MVG configuration exhibits a more pronounced pressure peak, demonstrating its role in intensifying local aerodynamic forces and enhancing flow mixing. This sharp pressure increase is critical for creating the conditions necessary to sustain an effective cooling layer. In the downstream region (*X/D* > 0), the configuration without the MVG maintains a relatively steady pressure distribution, with minimal oscillations and a gradual return to baseline values. Meanwhile, the configuration with the MVG exhibits minor fluctuations, likely due to residual effects of vortex structures. Despite these variations, the pressure distribution stabilizes further downstream as the vortices dissipate and integrate into the flow. Overall, the MVG significantly modifies the surface pressure distribution, introducing sharp variations upstream and amplifying pressure peaks at critical locations. These changes are crucial for enhancing vortex generation and improving flow mixing, ultimately improving thermal management and cooling performance. This analysis underscores the MVG’s effectiveness as a flow-control mechanism, demonstrating its potential to optimize aerodynamic and cooling applications.

As shown in Fig. 7c, the temperature along the stator blade exhibits a noticeable reduction of up to approximately 11% in the case of an MVG is utilized. Figure 7d illustrates the film cooling effectiveness along the normalized distance (X/D) for two scenarios: one without an MVG and the other with an MVG of 2.5 mm in height. The results clearly highlight the significant impact of the MVG in maintaining higher cooling effectiveness across the stator blade surface. At the injection point (*X/D* = 0), both scenarios exhibit very high film cooling effectiveness, nearly 100%, due to the strong protective layer formed by the cooling jet. However, as the flow progresses downstream, distinct differences become evident. In the absence of the MVG, the film cooling effectiveness drops sharply, likely due to insufficient mixing of the cooling air with the surrounding hot gas. This results in the rapid decay of the cooling layer. In contrast, the presence of the MVG leads to a more gradual decline in effectiveness. This improvement can be attributed to the vortices generated by the MVG, which enhance the mixing between the cooling air and the main flow, resulting in a more uniform cooling distribution. The differences between the two scenarios are particularly pronounced in the range of *X/D* = 0.05 to *X/D* = 0.15, where the MVG consistently provides higher cooling effectiveness. Even further downstream, beyond *X/D* = 0.2, the MVG configuration continues to perform better, though effectiveness decreases in both scenarios due to the natural spread and weakening of the cooling layer. Overall, the use of the *MVG* significantly enhances film cooling performance, demonstrating its potential to optimize cooling efficiency, particularly in applications requiring sustained, widespread cooling.

Figure 7e shows the enthalpy distribution along the blade surface for two scenarios: with and without the MVG. In both cases, enthalpy increases rapidly at the beginning of the blade, indicating significant heat transfer from the hot airflow to the blade surface. However, when MVG is present, the enthalpy values are lower. In the initial region, which covers the first 10% of the blade length, enthalpy decreases more dramatically in the presence of MVG. The greatest difference is observed near the injection point, where the enthalpy with MVG drops by 61%. This reduction suggests that the cooling jet mixes more effectively with the hot airflow, enhancing heat transfer. Beyond the first 10% of the blade length, the rate of increase in enthalpy slows down. This indicates a reduced temperature gradient between the blade surface and the surrounding airflow. The reduced gradient is attributed to greater temperature uniformity and less interaction between the cooling jet and the hot flow farther from the injection point. In the mid-section of the blade, the enthalpy difference between the two cases reaches 9.87%, demonstrating MVG’s effectiveness in improving heat dissipation. Adding MVG disrupts the thermal boundary layer, increases turbulence, and enhances mixing between the cooling jet and the hot gas. This mechanism significantly lowers the enthalpy at the blade surface, especially in the upstream region. Although this effect decreases downstream, the enthalpy difference remains significant throughout the blade length. As the distance from the injection point increases, the vortices’ influence also diminishes, leading to reduced heat dissipation efficiency in the downstream sections.

Figure 7f illustrates the static density behavior in two scenarios: without the MVG and with the MVG. In the case without the MVG, the graph shows a steady, downward trend, indicating stable flow behavior. The decrease in density suggests a drop in pressure and temperature at the inlet. In contrast, when the MVG is present, the graph exhibits oscillations. Initially, there’s a sudden spike in density, likely due to flow instabilities created by the MVG. These fluctuations lead to the formation of vortex structures and alter the flow pattern, significantly impacting subsequent cooling performance. Following this sharp increase, there is a rapid drop in density, driven by the breakdown of the flow structure and sudden pressure changes. After the injection point, both graphs show much lower density levels than before the injection, reflecting the cooling jet’s effects. Initially, both graphs rise quickly, reflecting the positive influence of the cooling jet on density and cooling performance. However, after about 7% of the distance, the trend becomes more uniform, showing only a very slight increase. This might be due to flow saturation or to limitations in the jet’s cooling effect. Furthermore, the MVG graph is positioned lower than the one without MVG, suggesting a negative impact on surface cooling. This reduced effectiveness can be attributed to changes in the flow pattern and increased disturbances in the cooling jet caused by the MVG. The data indicate that the MVG influences the density before the injection point, leading to fluctuations and improved mixing in the flow. However, after the injection point, these effects result in decreased cooling efficiency and lower density compared to the case without the MVG*.*

In Fig. 7g, the region before injection shows that the graph without the MVG exhibits stable laminar flow, characterized by low fluctuations and minimal pressure drop, leading to a consistent increase in cfRe. The low cfRe value indicates limited convective heat transfer, which is typical of laminar flow. Conversely, the MVG figure initially shows a rise, but then experiences a significant drop in cfRe due to the development of vortex instabilities and disturbances in the flow pattern. The introduction of the MVG increases the Reynolds number, leading to turbulent flow that enhances mixing but also introduces instability. Following the injection of the cooling jet, these disruptions result in a decrease in both velocity and pressure in its vicinity. The effective mixing of the cold jet with the warm fluid improves heat transfer and reduces temperature. As temperature decreases, the fluid’s density changes, influencing buoyancy forces and altering flow dynamics, ultimately increasing flow velocity. The colder, denser fluid from the jet moves downward or mixes with the hotter fluid, enhancing turbulence and further promoting mixing. With the reduction of instabilities and the return to a more stable condition, cfRe begins to rise again. This increase in cfRe reflects an improvement in the convective heat transfer coefficient, indicating that fluid mixing has become more effective. In the region following the injection, the MVG graph exhibits a steeper slope, indicating more substantial changes and heightened instabilities driven by the MVG’s influence on the flow and thermal mixing. The steeper slope also suggests that the flow is becoming increasingly turbulent, which can enhance the overall heat transfer efficiency in the system. This enhanced mixing not only stabilizes the flow in the mixing region but also helps maintain a higher heat transfer rate, ensuring that the colder fluid effectively replaces heat from the surface.

As shown in Fig. 7h, this interaction increases the Nusselt number after the injection of the cooling jet, indicating enhanced heat transfer. Enhanced mixing increases the contact area between the hot and cold fluids, enabling more effective convective heat transfer. The vortices also promote momentum exchange and help blend the temperature fields, resulting in a more consistent temperature difference between the two fluid streams. Moreover, introducing the cooling jet creates temperature gradients that lead to density variations. These changes in density affect buoyancy forces and alter the flow dynamics. The colder, denser fluid from the jet usually moves downward or mixes with the hotter fluid, further enhancing turbulence and mixing. This improved mixing not only increases the convective heat transfer coefficient but also stabilizes the flow in the mixing region, leading to a sustained increase in the Nusselt number. As the flow stabilizes downstream of the injection point, heat transfer remains consistently higher than in the absence of the cooling jet. Better mixing and fluid interactions ensure that heat from the surface is effectively replaced by the colder fluid, maintaining a higher heat transfer rate. The increased velocity in the mixing region further enhances the convective heat transfer coefficient, thereby increasing the Nusselt number and demonstrating the benefits of improved mixing and fluid interaction.

### Effect of micro vortex generator tip height on cooling efficiency

Increasing the tip height of MVGs enhances shear interaction between the main flow and the surface, leading to stronger streamwise vortices. These vortices enhance mixing and improve convective heat transfer by enhancing interaction between the coolant and the hot core flow, resulting in more effective surface cooling. However, above a certain height, stronger vortices can introduce excessive turbulence and deviation from the wall. This may cause boundary-layer separation, increase flow resistance, and lead to higher pressure losses, ultimately reducing overall system efficiency. Moreover, intense turbulent mixing can disturb the coolant film, lowering its effectiveness in protecting the surface. Therefore, the MVG tip height must be carefully optimized to strike a balance between improved mixing and heat transfer on one hand, and minimized pressure drop and flow instability on the other.

**Fig. 8:**
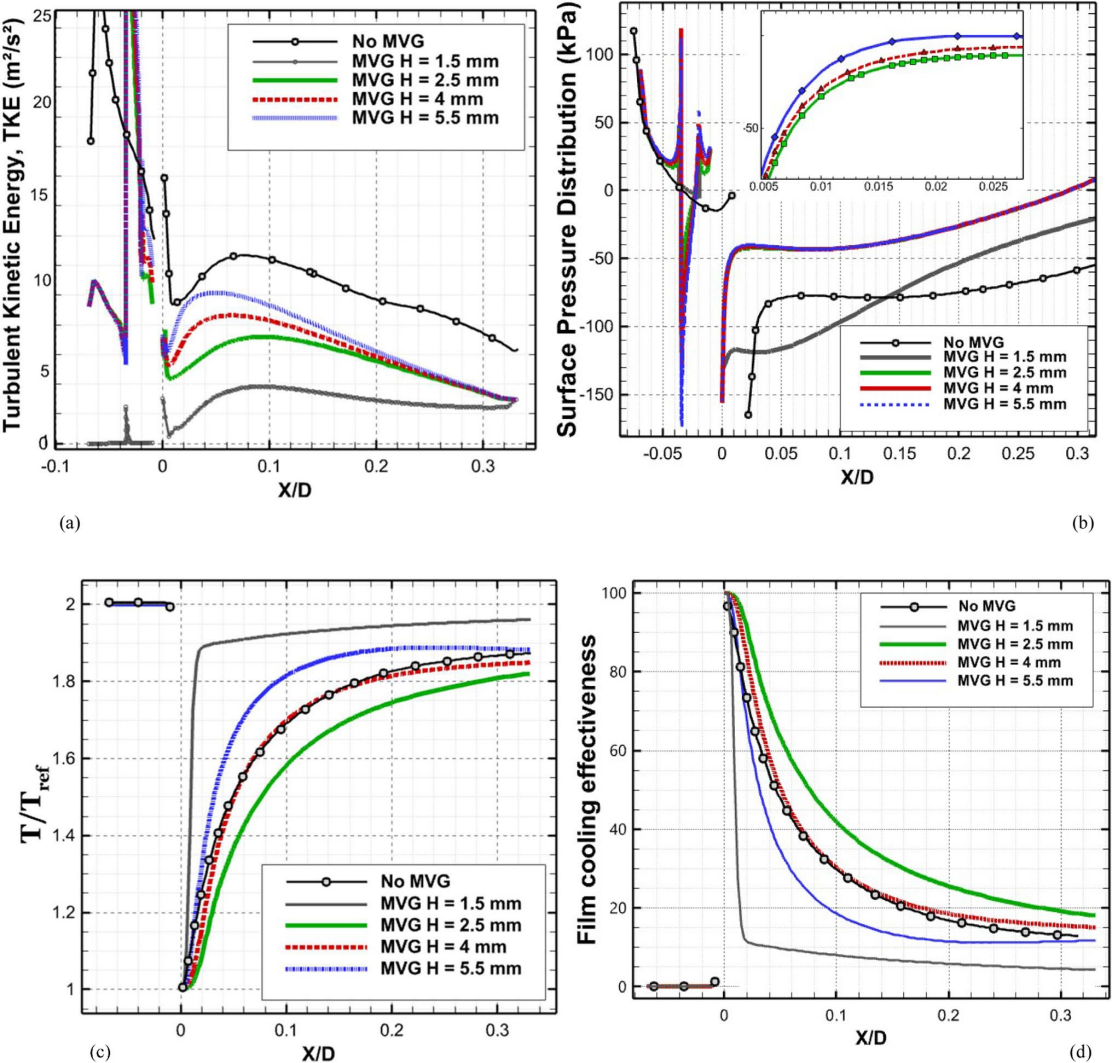

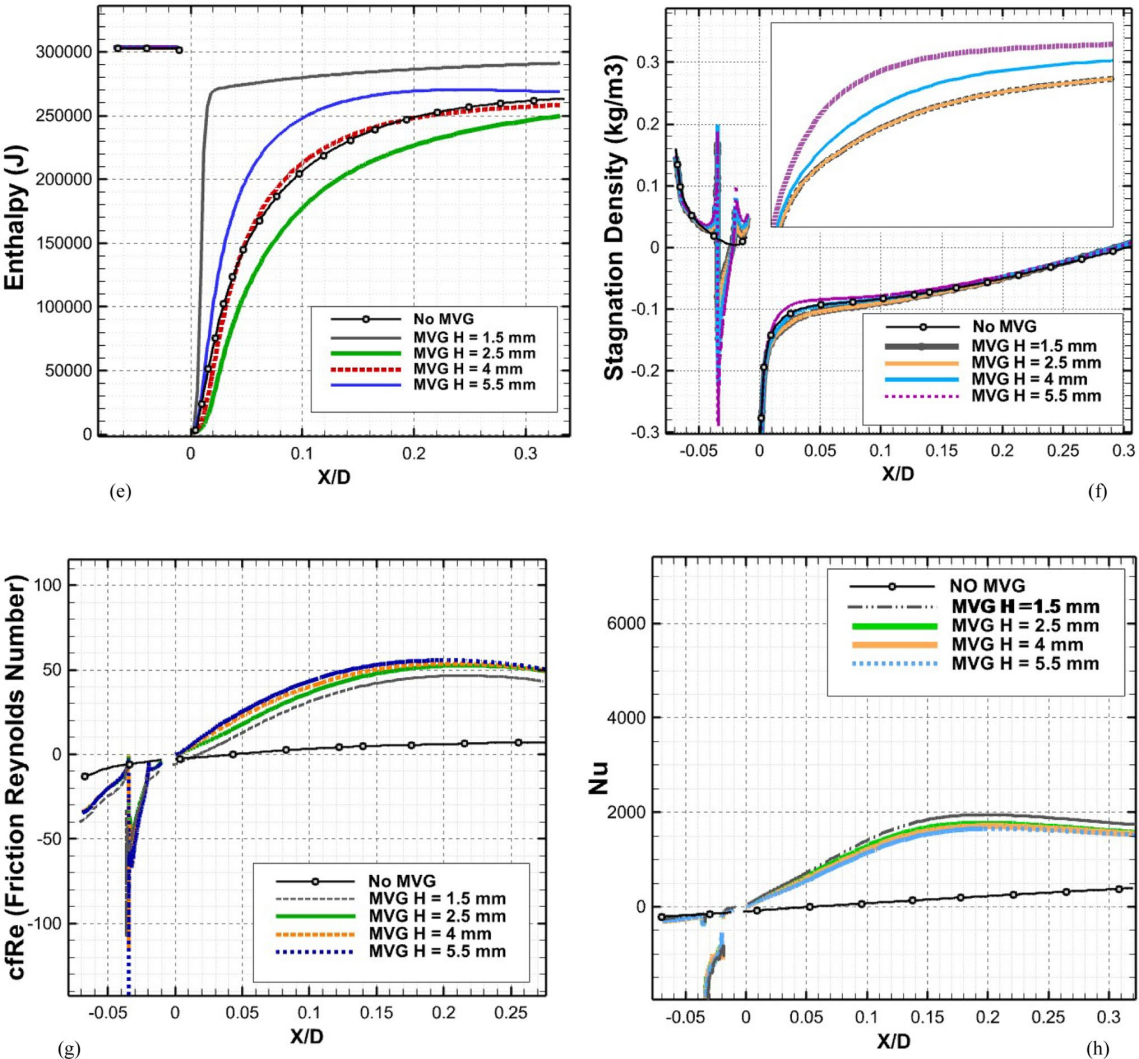
Surface cooling efficiency: comparison of conditions with and without MVG at heights of 2.5 mm, 4 mm, and 5.5 mm; (**a**) turbulence kinetic energy; (**b**) surface pressure; (**c**) temperature; and (**d**) film cooling effectiveness; (**e**) enthalpy; (**f**) stagnation density; (**g**) cfRe; (**h**) Nusselt number.

Figure 8a illustrates the variation of Turbulence Kinetic Energy (TKE) along the streamwise direction (*X/D*) for a baseline case without any control device and several cases with MVGs of different heights. From a fluid-dynamic and boundary-layer perspective, the behavior observed in the plot can be interpreted by considering how MVGs interact with the incoming boundary layer and modify the flow structure. In the absence of MVGs, the flow experiences a strong separation near the leading edge or the shock impingement region, resulting in a sharp spike in TKE. This is due to the intense shear and sudden velocity gradients present in the separated shear layer. The large peak in TKE observed before *X/D* = 0 is indicative of strong turbulent production due to uncontrolled shock-boundary-layer interaction. When MVGs are introduced, even with a minimal height (e.g., 1.5 mm), they begin to energize the boundary layer by introducing streamwise vortices. These vortices enhance momentum transfer from the core flow to the wall, effectively delaying or weakening flow separation. As a result, the sharp peak in TKE is significantly reduced in all MVG cases compared to the uncontrolled case. This indicates that MVGs help to stabilize the flow in the near field. However, as the MVG height increases, the strength of the induced vortices increases as well. While this promotes better mixing and improves separation control, it also introduces additional small-scale disturbances that contribute to turbulence. This is why we observe a gradual rise in TKE downstream (*X/D* > 0) as the MVG height increases from 2.5 to 5.5 mm. Essentially, higher MVGs inject more energy into the boundary layer, thereby sustaining turbulence farther downstream. There is a trade-off between flow control and turbulence augmentation: smaller MVGs (such as *H* = 2.5 mm) may be sufficient to suppress the initial separation without excessively increasing downstream TKE. In contrast, taller MVGs (*H* = 5.5 mm) offer stronger control but at the cost of higher turbulence intensity in the recovery region, which may not be desirable in applications where heat transfer or noise is a concern. From a thermal perspective, increased turbulence generally enhances convective heat transfer. Therefore, taller MVGs could be beneficial in cooling applications, despite the higher drag penalty. On the other hand, for aerodynamic surfaces where drag minimization is critical, a balance must be struck by choosing an MVG height that controls separation without introducing excessive turbulence. In summary, the plot reveals that MVGs effectively reduce peak turbulence caused by shock-induced separation, and their height plays a key role in determining the downstream turbulence characteristics. A moderate height appears to offer the best compromise between separation control and turbulence management.

Figure 8b presents the surface pressure distribution (in *kPa*) along the streamwise direction (*X/D*) for a baseline case without flow control and several cases with MVGs of varying heights. This plot offers valuable insights into how MVGs influence shock-boundary-layer interactions and pressure recovery by altering the near-wall flow. In the case without MVGs, represented by the black line with hollow circles, we observe a sharp drop in surface pressure near *X/D* = 0, followed by an oscillatory pattern and slow recovery downstream. This behavior is characteristic of shock-induced flow separation, where an adverse pressure gradient causes the boundary layer to separate from the surface. The large pressure gradient before and after the separation point leads to unsteady shock motion and the formation of a separated shear layer, reflected in strong pressure fluctuations and low wall pressure. When MVGs are introduced, even the smallest height (1.5 mm) alters this behavior significantly. The MVGs generate streamwise vortices that energize the near-wall region, promoting momentum exchange between the outer inviscid flow and the boundary layer. This momentum enhancement delays or weakens separation, resulting in a much smoother pressure distribution. In all MVG cases, the sharp pressure drop is reduced, and the flow recovers more quickly downstream. The inset in the figure, which zooms in on the recovery region (X/D ≈ 0.005–0.03), confirms the enhanced pressure recovery, especially for taller MVGs. As the MVG height increases from 1.5 to 5.5 mm, the strength of the generated vortices grows, improving their ability to control separation and restore pressure. Notably, the 5.5 mm MVG (blue dotted line) achieves the fastest and highest pressure recovery. However, this also suggests stronger flow disruption, which might not always be desirable depending on the thermal or aerodynamic application. From a thermal perspective, better pressure recovery and delayed separation help stabilize the flow and improve the predictability of heat transfer near the wall. However, if turbulence is too high—as is often the case with taller MVGs—it can increase thermal mixing and wall heat flux, which may be a concern in sensitive applications. Furthermore, from a boundary layer viewpoint, MVGs effectively thicken the boundary layer downstream by preventing early detachment. This shifts the flow behavior closer to an attached turbulent regime rather than a separated one, which is more stable and predictable in terms of pressure and thermal loading. In conclusion, MVGs significantly improve surface pressure distribution by suppressing separation and stabilizing the shock-induced flow. Taller MVGs are more effective in promoting pressure recovery but may introduce more turbulence and higher skin friction. Therefore, selecting an optimal MVG height requires balancing pressure recovery, separation control, and overall flow stability, depending on the specific goals of the thermal or aerodynamic system.

Figure 8c illustrates the variation of normalized temperature along the streamwise direction (*X/D*) for different MVG heights, as well as for the baseline case without MVGs. This data provides insights into how the presence and size of MVGs influence thermal transport near the surface in a high-speed or compressible flow regime. In the baseline case (no MVG), the temperature rises gradually downstream as the flow recovers from shock-induced separation. The slower temperature rise indicates weaker near-wall mixing and relatively poor thermal energy transport from the core flow toward the surface. This is expected, as the separated flow typically insulates the wall, reducing convective heat transfer in the near-wall region. When MVGs are introduced, the temperature response changes significantly. Even the smallest MVG (*H* = 1.5 mm) causes a noticeable jump in temperature shortly after *X/D* = 0. This is due to the generation of streamwise vortices, which enhance momentum and energy exchange between the high-speed outer flow and the low-momentum boundary layer. These vortices help reattach the flow earlier and promote stronger convective heat transfer, leading to a faster rise in surface temperature. As the MVG height increases, the strength and size of the generated vortices also increase. This results in more aggressive mixing, which transports thermal energy more efficiently toward the wall. Consequently, the surface temperature increases more rapidly and reaches higher values for taller MVGs. For example, the MVG with *H* = 5.5 mm shows the fastest and most significant temperature rise, while the 2.5 mm MVG shows a more moderate increase. This trend reflects the correlation between MVG-induced vortex strength and wall heat transfer. From a thermal-fluid perspective, these results suggest that MVGs act as passive enhancers of heat transfer. The stronger the induced vortices, the more they disrupt the thermal boundary layer, reduce its thickness, and increase the temperature gradient near the wall—all of which contribute to higher heat transfer rates. However, this comes with a trade-off: taller MVGs also tend to increase local wall shear stress and potentially elevate thermal loads, which might not be desirable in thermally sensitive components. Moreover, the observed differences among the curves highlight the sensitivity of the thermal field to MVG height. The 2.5 mm and 4 mm cases appear to offer a balance between improved temperature rise and controlled flow disturbance, which could be advantageous in designs requiring enhanced heat transfer without excessive thermal stress or drag penalties.

Figure 8d presents the variation in film cooling effectiveness along the streamwise direction (X/D) for different MVG heights, as well as for the baseline case without MVGs. Film cooling effectiveness is a key parameter in high-temperature applications, indicating how well the injected coolant protects the surface from the hot mainstream flow. In the no-MVG case, the effectiveness peaks sharply at the injection point but then drops off quickly as the coolant layer is rapidly eroded and mixed away by the high-speed hot flow. This behavior is typical in film cooling when there is no flow control; the coolant lacks enough momentum and coherence to remain attached to the wall, and it is quickly entrained into the main flow. With the addition of MVGs, this behavior changes significantly. MVGs generate streamwise vortices that modify the near-wall flow structure. While vortices can enhance momentum transfer and potentially increase surface heating (as seen in the previous temperature plot), they also play a critical role in anchoring and distributing the coolant layer more effectively along the surface. Among the MVG cases, *H* = 2.5 mm is the most effective configuration. It maintains a high cooling effectiveness over a longer downstream distance. This suggests that the vortices generated at this height are strong enough to promote lateral spreading of the coolant without causing excessive mixing with the hot mainstream. The result is a more stable and protective coolant film. The smallest MVG (1.5 mm) does not generate strong enough vortices to alter the flow field significantly, and in fact, its effectiveness in this case falls even faster than in the baseline. This may be due to weak circulation that disrupts the coolant jet without providing meaningful redistribution or protection. In contrast, the tallest MVG (*H* = 5.5 mm) generates very strong vortices that aggressively mix the coolant with the hot flow. While this improves thermal mixing and heat transfer, it reduces the surface-level protection provided by the coolant film. This is reflected in the sharp drop in effectiveness, especially just downstream of the injection location. The 4 mm MVG case performs moderately well, indicating a balance between control and mixing. However, it still underperforms compared to the 2.5 mm case, particularly in maintaining the coolant layer over longer distances. From a thermal-fluid perspective, effective film cooling depends not only on how well the coolant adheres to the wall but also on how the flow structures control its diffusion. Too little vortex strength leads to detachment and rapid decay; too much causes premature mixing with the hot flow. The 2.5 mm MVG appears to hit the “sweet spot,” providing enough flow control to stabilize the coolant layer without overly disrupting it. In summary, MVGs can either enhance or degrade film cooling depending on their geometry. While they promote heat transfer and boundary-layer control, their effectiveness in cooling applications depends on carefully tuning their height to preserve the protective coolant film. In this case, the 2.5 mm MVG achieves the best cooling performance by balancing coolant attachment and controlled dispersion.

Figure 8e shows the variation in total enthalpy along the streamwise direction (X/D) for different MVG heights, compared to the baseline case without flow control. Enthalpy, as a measure of thermal energy content, reflects both the temperature and the internal energy distribution in the boundary layer and is directly influenced by vortex-induced mixing and near-wall transport mechanisms. In the no-MVG case, the enthalpy increases steadily downstream due to the gradual thermal recovery following shock-induced separation. The weak momentum and thermal transport in the separated boundary layer result in a slower rise in surface enthalpy, as the core flow exchanges energy inefficiently with the near-wall region. With the introduction of MVGs, the flow behavior changes significantly. The presence of streamwise vortices enhances mixing between the high-enthalpy core flow and the cooler near-wall fluid. This promotes faster enthalpy recovery and reflects more efficient energy transfer. Even the smallest MVG (*H* = 1.5 mm) triggers an early jump in enthalpy, though the increase stabilizes quickly, indicating that the vortices are not strong enough to sustain deep mixing downstream. The MVG with *H* = 2.5 mm exhibits a more gradual and sustained rise in enthalpy, indicating effective yet balanced momentum and energy transport. This vortex strength appears optimal, re-energizing the boundary layer without over-disturbing it. The 4 mm MVG performs similarly, offering slightly faster recovery than the 2.5 mm case. By contrast, the 5.5 mm MVG leads to a much sharper rise in enthalpy, especially immediately downstream of the injection point. This is due to the stronger vortices penetrating deeper into the boundary layer, aggressively mixing the thermal layers. While this can improve heat transfer performance, it may also increase wall thermal loads, especially in sensitive applications. In summary, increasing MVG height enhances enthalpy recovery by strengthening vortex-induced mixing, but excessive vortex strength (as in the 5.5 mm case) can lead to unwanted thermal gradients. The 2.5–4 mm range appears to strike the best balance, offering effective energy transport while maintaining stable boundary layer behavior.

Figure 8f illustrates the streamwise variation of stagnation density for different MVG heights compared to the uncontrolled baseline. Stagnation density reflects the combined effects of pressure and temperature recovery and is sensitive to shock-boundary-layer interactions and flow-control strategies. In the no-MVG case, a sharp drop in stagnation density occurs around *X/D* = 0, indicating strong shock-induced separation and energy loss in the boundary layer. This is followed by a slow recovery downstream, suggesting limited mixing and inefficient momentum and energy transport toward the wall. With MVGs introduced, the flow response changes significantly due to the formation of streamwise vortices that re-energize the boundary layer and promote mixing. All MVG cases exhibit smoother transitions and faster recovery of stagnation density compared to the baseline. The inset plot highlights these differences. The 1.5 mm MVG produces only a slight improvement, indicating weak vortex strength and limited influence on the separated flow. The 2.5 mm and 4 mm MVGs generate stronger, more organized vortices that enhance momentum and energy exchange with the core flow. As a result, stagnation density recovers more steadily, reflecting a healthier, reattached boundary layer with better preservation of total pressure and thermal energy. The 5.5 mm MVG causes the most rapid and pronounced recovery. However, this is a result of more aggressive mixing, which can lead to increased thermal and pressure gradients. While beneficial for restoring flow uniformity, such intense vortex activity may increase surface loading and thermal stress. In short, MVGs mitigate the adverse effects of shock-induced separation and improve stagnation density recovery. The heights of 2.5–4 mm offer a good balance between control effectiveness and flow stability, while 5.5 mm induces stronger but potentially excessive disturbances.

Figure 8g presents the variation of the friction Reynolds number (cfRe) along the streamwise direction (*X/D*) for different MVG heights, as well as the baseline case without MVGs. This parameter represents the product of skin friction and the Reynolds number, serving as an indicator of wall shear and momentum transport within the boundary layer. In the no-MVG case, cfRe remains close to zero throughout, with a small negative dip near *X/D* = 0. This behavior reflects boundary-layer separation caused by the shock interaction, characterized by reversed flow and low wall shear stress. The absence of reattachment keeps the wall shear minimal and recovery slow. With MVGs applied, the flow behavior changes considerably. The generation of streamwise vortices enhances near-wall momentum, leading to earlier reattachment and an increase in cfRe downstream. The initial negative spike near the shock location becomes less severe with MVGs, and cfRe quickly transitions to positive values, signaling restored wall shear. Among the MVG configurations:

- The 1.5 mm MVG shows modest improvement in cfRe, suggesting limited vortex strength and weaker control.
- The 2.5 mm and 4 mm MVGs result in smoother, sustained increases in cfRe, indicating strong and stable vortex-induced reattachment with enhanced shear development along the surface.
- The 5.5 mm MVG produces the highest peak in cfRe but also shows a sharper gradient. This reflects more aggressive momentum injection and possibly higher local wall stress, which can be beneficial for mixing but might introduce thermal or mechanical penalties in sensitive zones.

Overall, increasing MVG height enhances wall shear by strengthening the vortex–boundary-layer interaction. The 2.5–4 mm range achieves effective recovery without overshooting shear levels, while the tallest MVG yields maximum cfRe at the cost of more intense surface loading.

Figure 8h shows the streamwise variation of the Nusselt number (*Nu*) for different MVG heights, compared to the baseline case without MVGs. The Nusselt number quantifies convective heat transfer between the wall and the flow and is directly influenced by the boundary layer’s structure and behavior. In the no-MVG case, Nu remains near zero throughout, reflecting weak convective transport due to shock-induced separation and a poorly energized boundary layer. The lack of near-wall momentum impedes efficient heat transfer, resulting in poor thermal performance. With MVGs introduced, the flow structure is significantly modified. The streamwise vortices generated by the MVGs re-energize the boundary layer, enhancing wall-normal transport and thinning the thermal boundary layer. As a result, the Nusselt number rises sharply after the interaction point, indicating a substantial increase in convective heat transfer. Comparing MVG heights:

- The 1.5 mm MVG shows the highest peak *Nu*. Its vortices appear strong enough to promote early and sharp heat transfer without excessively disturbing the wall layer.
- The 2.5 mm and 4 mm MVGs offer slightly lower but broader peaks, suggesting stable and sustained thermal enhancement downstream. These configurations effectively balance mixing and boundary layer recovery.
- The 5.5 mm MVG leads to the most extended thermal development but with a flatter Nu profile. This implies strong mixing, which, while enhancing heat transfer, may lead to earlier thermal saturation or a more uniform wall temperature downstream.

Overall, increasing MVG height strengthens vortex-induced convective heat transfer, but the effect saturates beyond a certain point. The 1.5–4 mm range delivers high local Nusselt numbers with controlled thermal loading, while the 5.5 mm height promotes distributed but less intense heat transfer. The choice depends on whether peak heat flux or broader thermal coverage is desired.

### Effect of micro vortex generator angle and slope variation

**Fig. 9:**
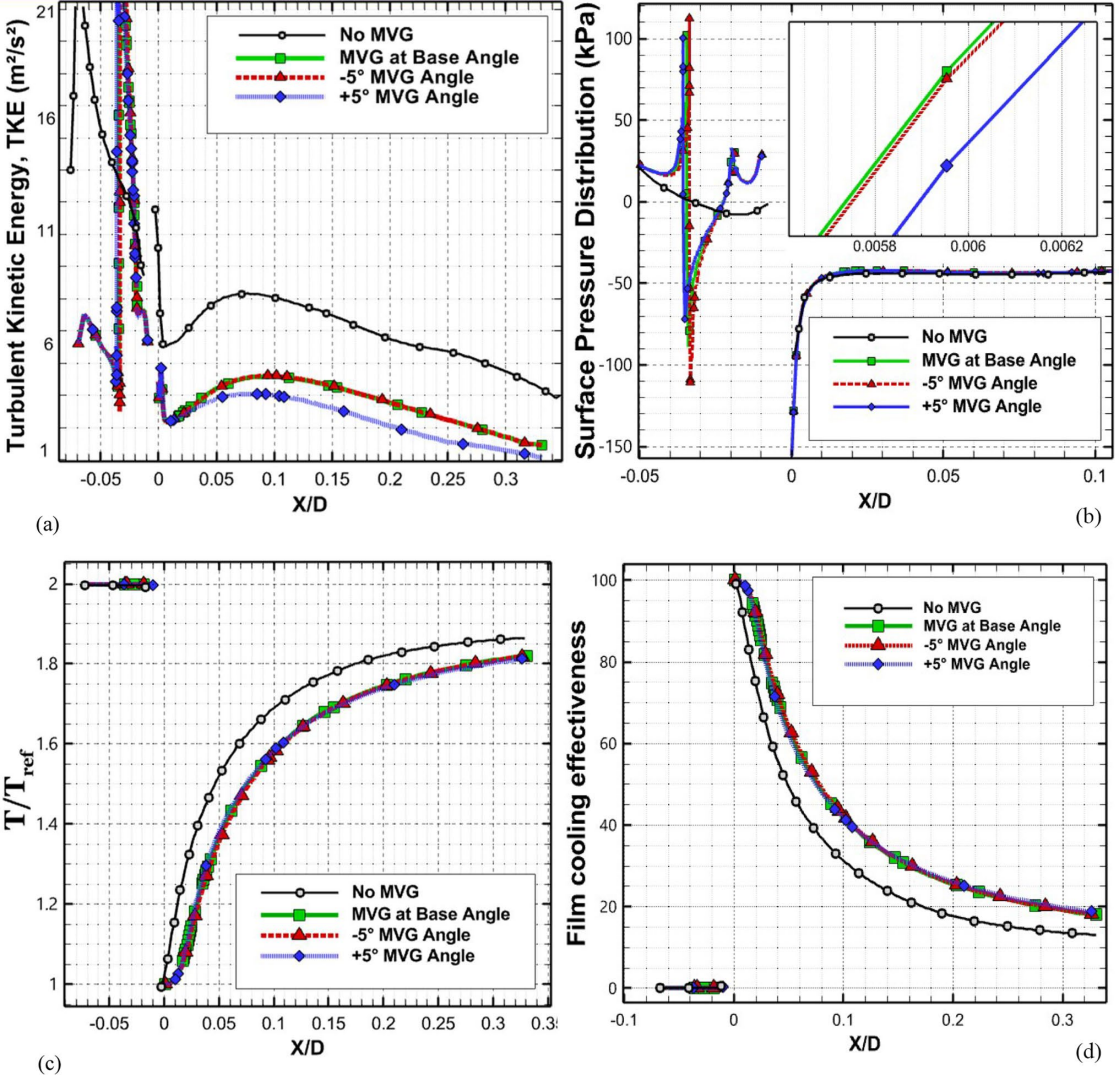

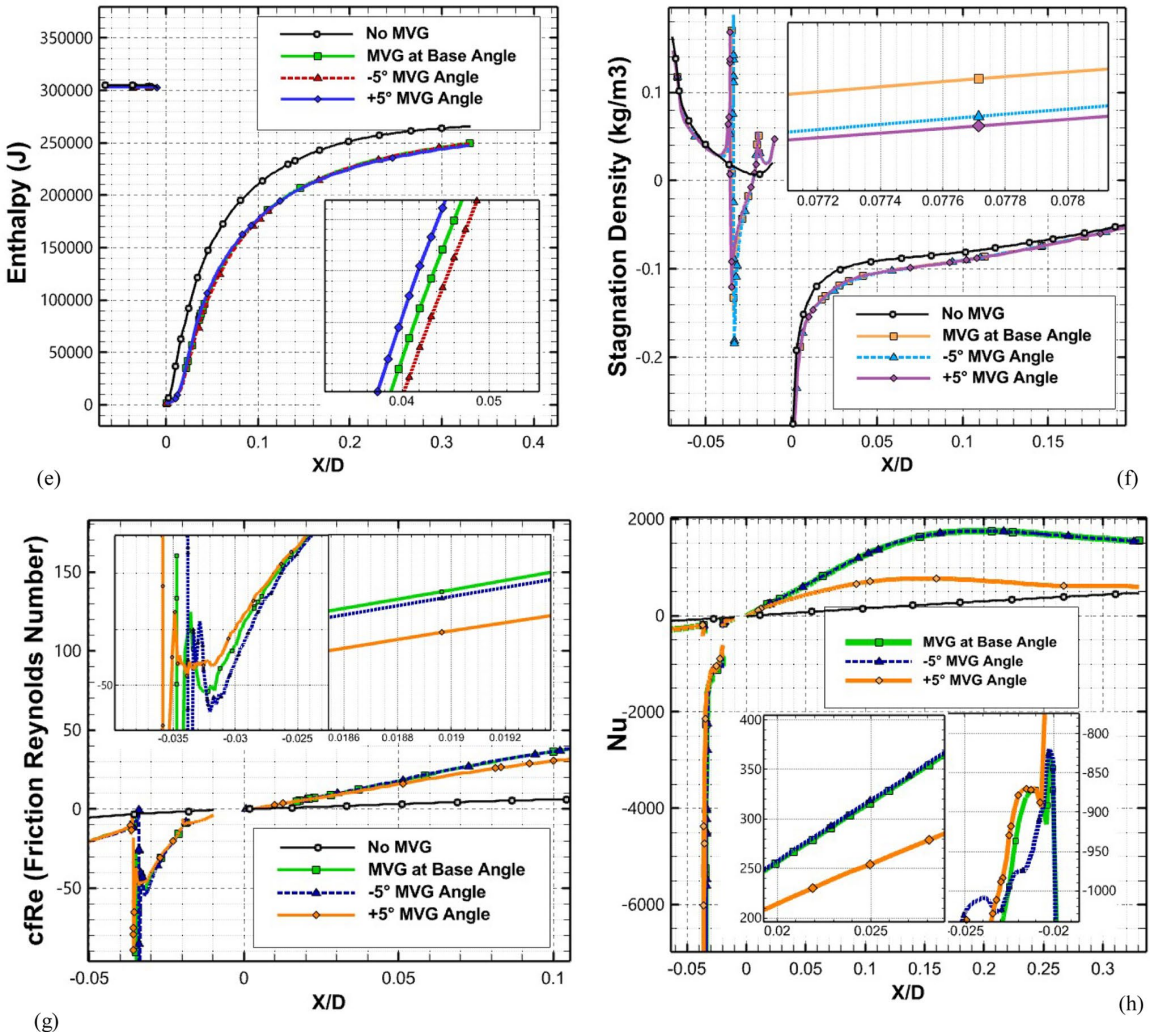
Surface cooling efficiency: comparison of the baseline case and MVGs with apex angle variations of ± 5 degrees; (**a**) turbulence kinetic energy; (**b**) surface pressure; (**c**) temperature; and (**d**) film cooling effectiveness; (**e**) enthalpy; (**f**) stagnation density; (**g**) cfRe ; (**h**) Nusselt number.

Based on Fig. 9a, for the cases with MVGs, the turbulence kinetic energy (TKE) initially starts much lower than the baseline (no-MVG) case. However, after a short distance, the TKE increases sharply, surpassing the baseline by a significant margin. This increase is attributed to the vortices created by the MVGs, which promote turbulence and flow mixing, leading to higher TKE compared to the smoother flow in the baseline case. Once the coolant jet is introduced, starting from *X* = 0, the TKE for the MVG cases drops significantly, becoming much lower than the baseline case. The lowest *TKE* is observed in the + 5° MVG configuration. This can be attributed to the fact that the + 5° configuration generates weaker vortices compared to the other MVG configurations, resulting in less turbulence and lower TKE. The weaker vortices reduce the turbulent mixing and energy dissipation, leading to the lowest kinetic energy in the + 5° case.

In Fig. 9b, before the coolant jet is injected and while the hot air flow interacts with the MVGs at three different angles, the pressure initially increases sharply compared to the baseline (no MVG) curve. This increase is due to the formation of localized vortices generated by the MVGs. These vortices temporarily disrupt the flow, causing a rapid rise in pressure near the leading edge. However, shortly after, the pressure drops sharply. This decrease occurs because the vortices cause turbulence and disrupt the boundary layer, resulting in a loss of pressure further downstream. After the coolant jet is injected (starting from *X* = 0), the pressure levels in all configurations become very similar, with only a small difference between them. The pressures in all cases are almost the same, except for a slight reduction in the + 5° case, which is minimal. This indicates that the coolant jet effectively blends the flow and reduces the impact of the MVG configurations, resulting in nearly identical pressure distributions. In summary, while the MVGs have a noticeable effect on the pressure profile before the jet injection, their influence diminishes after the jet is introduced. The coolant jet enhances flow mixing and reduces the differences between the MVG configurations, resulting in nearly identical pressure distributions, with only a slight difference in the + 5° angle case.

Based on Fig. 9c, d, the presence of MVGs at all three angles (baseline, + 5°, and − 5°) leads to a reduction in the stator surface temperature by up to 20% compared to the cooling case without MVGs. This temperature drop occurs because the MVGs enhance flow mixing and heat transfer, improving the effectiveness of the cooling film and the airflow over the surface. However, it is important to note that the change in the apex angle, whether increasing by + 5° or decreasing by − 5°, does not significantly affect the temperature reduction. In other words, altering the angle of the MVGs has no significant impact on cooling performance or surface temperature. This could be because the vortex formation and flow mixing are similar for both + 5° and − 5° configurations, meaning the angle changes within this range don’t have a substantial effect. Additionally, the cooling system might be sufficiently optimized that small variations in the MVG angle do not result in a noticeable difference in stator surface cooling.

As shown in Fig. 9e, f the enthalpy and stagnation density curves for all MVG configurations are significantly lower than the no-MVG case, indicating effective enthalpy reduction. The 5-degree angle variation has a minimal impact on enthalpy, suggesting a negligible effect on overall thermal performance.

Figure 9g, illustrates that after injection, the MVG curves exhibit a notable increase in cfRe, reflecting heightened turbulence and mixing resulting from the interaction of the cooling jet with the flow. This rise is linear and significantly higher than the stable, lower cfRe seen in the no-MVG case. This behavior underscores the MVG’s role in enhancing convective heat transfer by promoting increased flow velocity and facilitating momentum exchange within the mixing region.

As shown in Fig. 9h, in the no-MVG case, the slow, linear rise in the Nusselt number is due to the gradual development of the thermal boundary layer and limited heat transfer in a less turbulent flow. On the other hand, the MVG with a higher 5-degree angle creates stronger turbulence and higher thermal mixing in the early stages, resulting in a nonlinear increase in Nusselt number. However, this effect diminishes midway along the blade as the temperature difference between the jet and the main flow decreases and the vortex structures weaken, resulting in a drop in the Nusselt number. The similar behavior of the initial MVG and the one with a smaller 5-degree angle indicates that both achieve comparable levels of mixing and heat transfer enhancement. Toward the blade’s end, the curves stabilize due to balanced temperatures and a steady turbulent flow. The higher Nusselt numbers of the initial MVG and the smaller-angle MVG compared to the larger-angle MVG can be attributed to optimized vortex structures and more efficient mixing. In contrast, the larger-angle MVG causes excessive turbulence early on, leading to higher pressure losses and increased energy dissipation, which further lowers heat transfer efficiency downstream.

### Effect of micro vortex generator position relative to coolant jet injection location

**Fig. 10:**
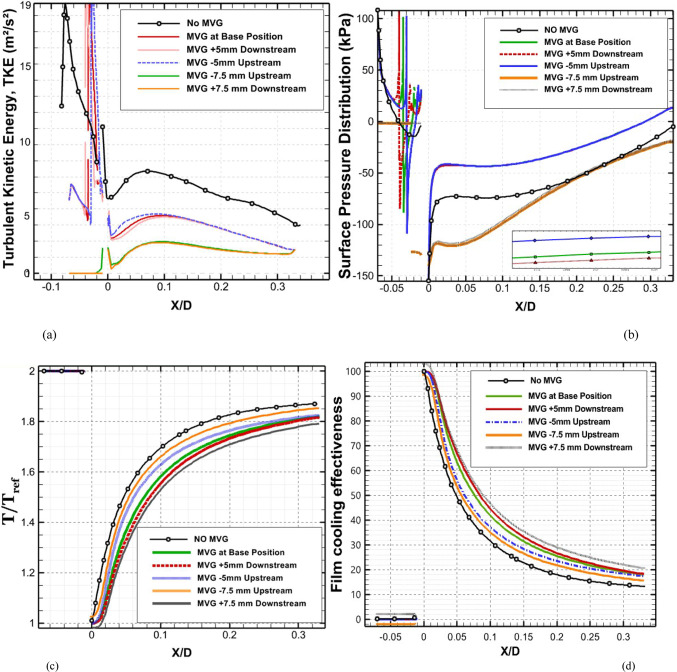

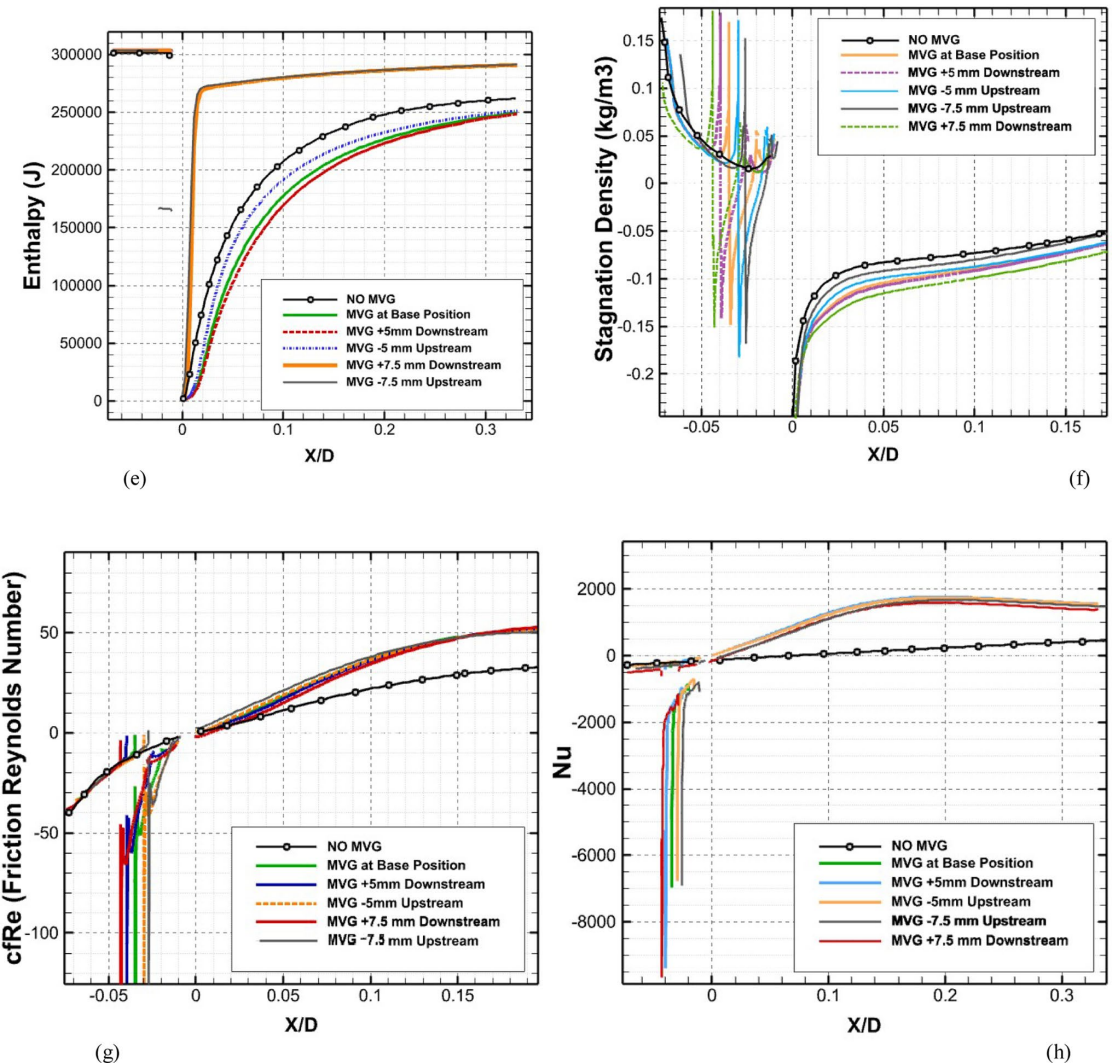
Surface cooling efficiency: comparison of the baseline case and MVGs positioned 5 mm farther and 5 mm closer to the coolant jet injection point; (**a**) turbulence kinetic energy; (**b**) surface pressure; (**c**) temperature; and (**d**) film cooling effectiveness; (**e**) enthalpy; (**f**) stagnation density; (**g**) cfRe ; (**h**) Nusselt number.

Figure 10a shows how the streamwise positioning of an MVG affects the distribution of turbulence kinetic energy (TKE) compared to the baseline case without MVGs. TKE is a direct measure of the intensity of turbulent fluctuations, and its variation reflects how effectively the MVG modifies the flow structure, particularly the boundary layer and shock interaction region. In the no-MVG case, the TKE sharply peaks near *X/D* ≈ 0 due to strong shock-induced separation and shear layer formation. The high TKE in this region indicates unstable flow and poor control over boundary layer behavior. When an MVG is introduced at the base position, TKE is significantly reduced in the shock-impingement zone and exhibits a controlled rise downstream. This indicates that the MVG successfully re-energizes the boundary layer and stabilizes the shock–boundary-layer interaction, thereby reducing turbulence generation. Shifting the MVG 5 mm or 7.5 mm upstream weakens this effect. The green and blue curves show a delayed and reduced TKE response, implying that the MVG-generated vortices dissipate before reaching the separation region. As a result, they fail to interact effectively with the shock or reattach the boundary layer promptly. Conversely, placing the MVG at 5 mm or 7.5 mm downstream (indicated by the orange and blue dashed lines) also diminishes its effectiveness. The vortices are generated too late—after the critical interaction region—leading to minimal suppression of the initial TKE spike. This results in weak separation control and less turbulent stabilization. Overall, the base position proves to be the most effective, as it aligns vortex generation with the region of the strongest adverse pressure gradient. Proper alignment ensures that the vortices are active where needed—just upstream of separation—thus reducing turbulence generation and improving flow stability.

Figure 10b illustrates the effect of MVG placement along the streamwise direction on the surface pressure distribution, compared to the uncontrolled case. Pressure behavior in this region is directly linked to shock–boundary layer interaction and flow separation. In the no-MVG case, a sharp drop in surface pressure occurs near *X/D* ≈ 0, followed by unsteady recovery downstream. This pattern is typical of shock-induced boundary-layer separation, where an adverse pressure gradient leads to reversed flow and unstable shear layers. Placing the MVG at the base position significantly improves the pressure profile. The initial pressure drop is moderated, and the recovery becomes faster and smoother. This indicates that the streamwise vortices generated by the MVG interact effectively with the boundary layer just upstream of separation, improving its resistance to adverse pressure gradients and delaying or suppressing separation. When the MVG is moved upstream by 5 mm or 7.5 mm, the effectiveness decreases. The orange and blue curves show less favorable pressure recovery. In these cases, the vortices form too early and dissipate before reaching the critical interaction zone. As a result, the pressure drop remains severe, and the flow separation is less controlled. Shifting the MVG downstream by 5 mm or 7.5 mm also weakens its impact. Here, the vortices are generated too late—after separation has already begun. This is evident in the purple and red curves, which closely track the no-MVG case in the near-shock region. Pressure recovery improves only farther downstream, where the vortices eventually interact with the boundary layer. Among all configurations, the base position remains optimal. It aligns the vortex formation with the region of highest adverse pressure gradient, ensuring that the boundary layer receives the necessary momentum support right where it’s most needed. In short, proper MVG placement is critical. Moving it upstream or downstream—even slightly—reduces its ability to stabilize the boundary layer and improve surface pressure recovery. The results confirm that targeted alignment of vortex generation with the separation-prone region is essential for effective flow control.

Figure 10c illustrates how the streamwise position of the MVG affects surface-temperature recovery relative to the case without flow control. Surface-temperature behavior in this context is governed by how well the boundary layer remains attached and by how efficiently thermal energy is transported toward the wall. In the no-MVG case, the temperature rises gradually downstream, reflecting weak near-wall mixing and inefficient convective heat transfer due to shock-induced separation. The boundary layer remains thick and insulated, limiting thermal transport from the core flow to the surface. Introducing an MVG at the base position significantly enhances heat transfer. The temperature rises faster and reaches lower asymptotic values, suggesting that the streamwise vortices energize the boundary layer, reduce separation, and thin the thermal boundary layer. This improves wall-normal heat exchange and enhances local cooling effectiveness. Moving the MVG upstream by 5 mm or 7.5 mm slightly reduces this benefit. In these cases (blue and orange curves), the vortices form earlier but decay before reaching the shock-interaction zone, leading to weaker mixing where it is most needed. As a result, the rate of temperature increase is slower, and surface heating remains relatively high. Shifting the MVG downstream by 5 mm or 7.5 mm (red and purple curves) also diminishes thermal control. Since vortex generation is delayed, the boundary layer remains under-energized in the early interaction region, leading to less effective heat transfer initially and higher surface temperatures throughout. Among all configurations, the base position achieves the best thermal performance, enabling early boundary layer reattachment and more efficient heat removal through enhanced convective transport. In summary, aligning vortex formation with the critical shock–boundary layer interaction region is key to maximizing surface cooling. Both upstream and downstream offsets reduce the MVG’s beneficial impact on temperature control.

Figure 10d shows the variation in film cooling effectiveness along the streamwise direction (X/D) for different MVG positions, compared to the uncontrolled case. Film cooling effectiveness measures how well the coolant protects the surface from the hot mainstream flow and is closely linked to the boundary-layer structure and to how well the coolant remains attached to the wall. In the no-MVG case, effectiveness peaks sharply near the injection point but drops off quickly downstream. This steep decline indicates that the coolant film is quickly eroded and lifted off by the high-speed mainstream, due to the lack of flow control or vortex-induced stabilization. Adding an MVG significantly changes this behavior. When placed in the base position, the MVG generates strong streamwise vortices that enhance the lateral spreading of the coolant and help it remain attached to the wall. This delays the breakdown of the coolant layer, resulting in higher effectiveness over a longer distance. When the MVG is shifted 5 mm or 7.5 mm upstream, its performance diminishes. Earlier vortex generation weakens the structures before they reach the region of primary coolant interaction, making them less effective at stabilizing the film. This is reflected in the slightly faster decay in effectiveness. Shifting the MVG 5 mm or 7.5 mm downstream also reduces performance. In these cases, the vortices form too late, after much of the coolant has already detached or mixed with the hot core flow. As a result, they are less able to organize and spread the film near the wall, leading to lower effectiveness, especially in the near-field. Among all configurations, the base position provides the most stable and effective coolant film. It aligns vortex generation with the initial coolant injection zone, maximizing momentum exchange and anchoring the coolant layer close to the wall. Placing the MVG at the base position provides the best cooling performance, as it ensures that vortex generation occurs in the region where coolant stabilization is most critical. This alignment helps maintain higher effectiveness over a longer distance, whereas upstream or downstream displacements weaken the interaction and reduce the cooling film’s stability and coverage.

Figure 10e shows how enthalpy varies along the streamwise direction (*X/D*) for different MVG positions, as well as the case without MVGs. Enthalpy reflects the thermal energy content of the flow and is strongly influenced by shock–boundary layer interaction, vortex-induced mixing, and heat transfer to the wall. In the no-MVG case, enthalpy increases gradually downstream as the boundary layer recovers from shock-induced separation. The rise is relatively slow, indicating limited mixing and weak near-wall momentum and energy exchange. When an MVG is placed at the base position, it enhances near-wall transport by generating streamwise vortices that energize the boundary layer. These vortices promote earlier reattachment and stronger convective mixing, which accelerates enthalpy growth. The green curve shows clear improvement over the baseline. Shifting the MVG 5 mm upstream maintains good performance, although the earlier vortex generation causes slightly reduced impact in the critical region near the shock. In contrast, placing the MVG 5 mm downstream weakens the effect, since the vortices form too late to influence the separated flow effectively. This leads to slower enthalpy recovery compared to the base position. The 7.5 mm upstream placement produces an abnormally steep and early enthalpy rise, which may indicate premature vortex breakdown or numerical anomalies. The 7.5 mm downstream case also shows moderate performance but fails to match the controlled, gradual recovery observed at the base or − 5 mm positions. Positioning the MVG near the base ensures that the vortex structures are strongest where mixing is most needed—just upstream of the separation zone—leading to smoother, more effective enthalpy recovery along the wall. Shifting the MVG too far upstream or downstream reduces its ability to control the thermal boundary layer efficiently.

Figure 10f shows how stagnation density evolves along the streamwise direction (*X/D*) for various MVG positions, compared to the uncontrolled baseline. Stagnation density reflects both momentum and thermal recovery and is a sensitive indicator of shock–boundary layer interaction and overall flow energy retention. In the no-MVG case, the stagnation density drops significantly near the shock-interaction region (around X/D = 0) and then gradually recovers downstream. This behavior is characteristic of shock-induced separation, in which energy losses and disrupted boundary-layer structure lead to reduced total density near the wall. Placing the MVG at the base position (black solid line) improves the recovery of stagnation density. The streamwise vortices enhance near-wall mixing and momentum transfer, thereby supporting earlier boundary-layer reattachment and smoother total-pressure recovery. When the MVG is moved 5 mm upstream (blue dashed line), the vortices begin interacting with the flow too early and partially dissipate before reaching the key separation zone. This results in a weaker effect on the recovery of stagnation density. In the 7.5 mm upstream case (pink solid line), the early vortex formation appears to generate unstable or excessive disturbances, leading to unsteady variations and poor recovery. This may be due to premature vortex breakdown or misalignment with the separation region. On the other hand, downstream shifts of the MVG (green and purple lines) show more moderate improvements. While some positive influence is observed, the vortex structures form too late to prevent initial separation and associated energy loss. As a result, stagnation density recovers slowly and remains below that of the base-position case. Overall, locating the MVG near the base ensures that the vortex activity aligns closely with the shock-affected region, thereby preserving a greater portion of the total flow energy and promoting more effective downstream recovery. Upstream or downstream displacements reduce this benefit by misaligning the vortex–boundary layer interaction.

Figure 10g shows the variation of the friction Reynolds number (cfRe) along the streamwise direction (*X/D*) for different MVG positions. cfRe is a measure of wall shear stress scaled by local Reynolds number and is a direct indicator of boundary layer attachment and momentum transfer near the wall. In the no-MVG case, cfRe drops to negative values near *X/D* = 0, indicating boundary-layer separation due to shock interaction. The negative region reflects reversed flow near the surface. The recovery is slow, and cfRe remains low downstream due to the weakened boundary layer and lack of reattachment. With an MVG placed at the base position, the curve shows a faster transition from negative to positive cfRe, followed by a sustained rise. This indicates that the MVG-generated vortices reinforce the near-wall region, suppress separation, and accelerate reattachment, restoring wall shear more effectively. Shifting the MVG 5 mm upstream slightly reduces its impact. Although the vortices still help the flow reattach, they begin to dissipate before the separation zone, reducing recovery efficiency compared to the base location. The 5 mm and 7.5 mm downstream cases show similar trends: the cfRe increase is delayed since vortex generation occurs too late to prevent initial separation. While the boundary layer eventually reattaches, it does so farther downstream, reducing the area of high shear. The 7.5 mm upstream case shows inconsistent cfRe behavior near the shock, likely due to premature vortex decay or unsteady interaction, which weakens its ability to maintain stable wall shear in the critical region. Overall, the base-position MVG delivers the most favorable wall shear profile, with earlier reattachment and stronger cfRe growth. This highlights the importance of aligning vortex formation with the region of the peak adverse pressure gradient to maximize boundary-layer control.

Figure 10h shows the variation of the Nusselt number (*Nu*) along the streamwise direction (*X/D*) for different MVG positions, compared to the no-MVG case. *Nu* is a measure of convective heat transfer at the wall, reflecting how effectively the flow transports thermal energy from the core to the surface. In the no-MVG case, *Nu* remains close to zero throughout, indicating poor convective heat transfer due to shock-induced separation. The boundary layer remains thick and insulated, with minimal wall-normal thermal exchange. Introducing an MVG significantly improves heat transfer by generating streamwise vortices that energize the boundary layer and thin the thermal sublayer. When placed at the base position, the MVG enhances *Nu* consistently downstream of the interaction region. The green curve shows a sharp rise, reflecting improved wall cooling as the flow reattaches and convective transport increases. Moving the MVG 5 mm upstream also boosts *Nu* but with slightly diminished effectiveness. The vortices form too early and lose strength before reaching the critical zone of adverse pressure gradient, reducing their ability to control separation and enhance heat transfer near the wall. The downstream placements (5 mm and 7.5 mm) show strong *Nu* peaks, particularly the 7.5 mm downstream case (red curve), which maintains the highest heat transfer rate. In this position, the MVG-generated vortices remain strong and act closer to the reattachment zone, aggressively thinning the thermal boundary layer and increasing local heat flux. The 7.5 mm upstream placement (orange curve) shows an early spike. Still, its effect tapers off more quickly downstream, indicating that premature vortex activity may not sustain high thermal transport through the critical region. While all MVG configurations improve *Nu* compared to the baseline, the base and downstream positions are most effective in maintaining elevated heat transfer over the target region. Their vortex structures interact more favorably with the recovering boundary layer, enabling stronger, more persistent convective heat transfer at the wall.

To further interpret the wall heat transfer behavior, the Nusselt number distributions in Figs. 7h, 8h, 9h, and 10h show that the local Nu values become negative in some regions while remaining positive elsewhere. A negative Nusselt number indicates a reversal of the net wall heat flux toward the near-wall fluid, meaning that the wall releases heat to the fluid even as it continues to receive energy from the mainstream. This occurs when the heat transferred from the wall to the adjacent fluid exceeds the heat entering from the mainstream flow or by conduction through the wall. Under these conditions, the local wall heat flux becomes negative, as defined by Eqs. (18) and (19)^54,55^. This behavior is particularly evident in areas where strong vortical structures form due to the interaction between the hot mainstream flow and the MVG^3,56^. These vortices enhance local mixing and generate complex temperature gradients near the wall. In some locations, the temperature of the fluid adjacent to the surface becomes lower than the wall temperature (${T}_{\mathrm{fluid,local}}<{T}_{w}<{T}_{\infty }$), causing the wall to transfer heat to the fluid while still absorbing energy from the main flow. In cases with MVGs, this effect is more pronounced because the induced vortices amplify local thermal nonuniformities and promote heat-flux reversal^56^. The coexistence of positive and negative Nu regions reveals how vortex interactions locally alter the direction and magnitude of heat flux along the wall, reflecting the local dominance of vortex-driven convection over mainstream heating and illustrating the complex thermal balance near the wall.

**Fig. 11:**
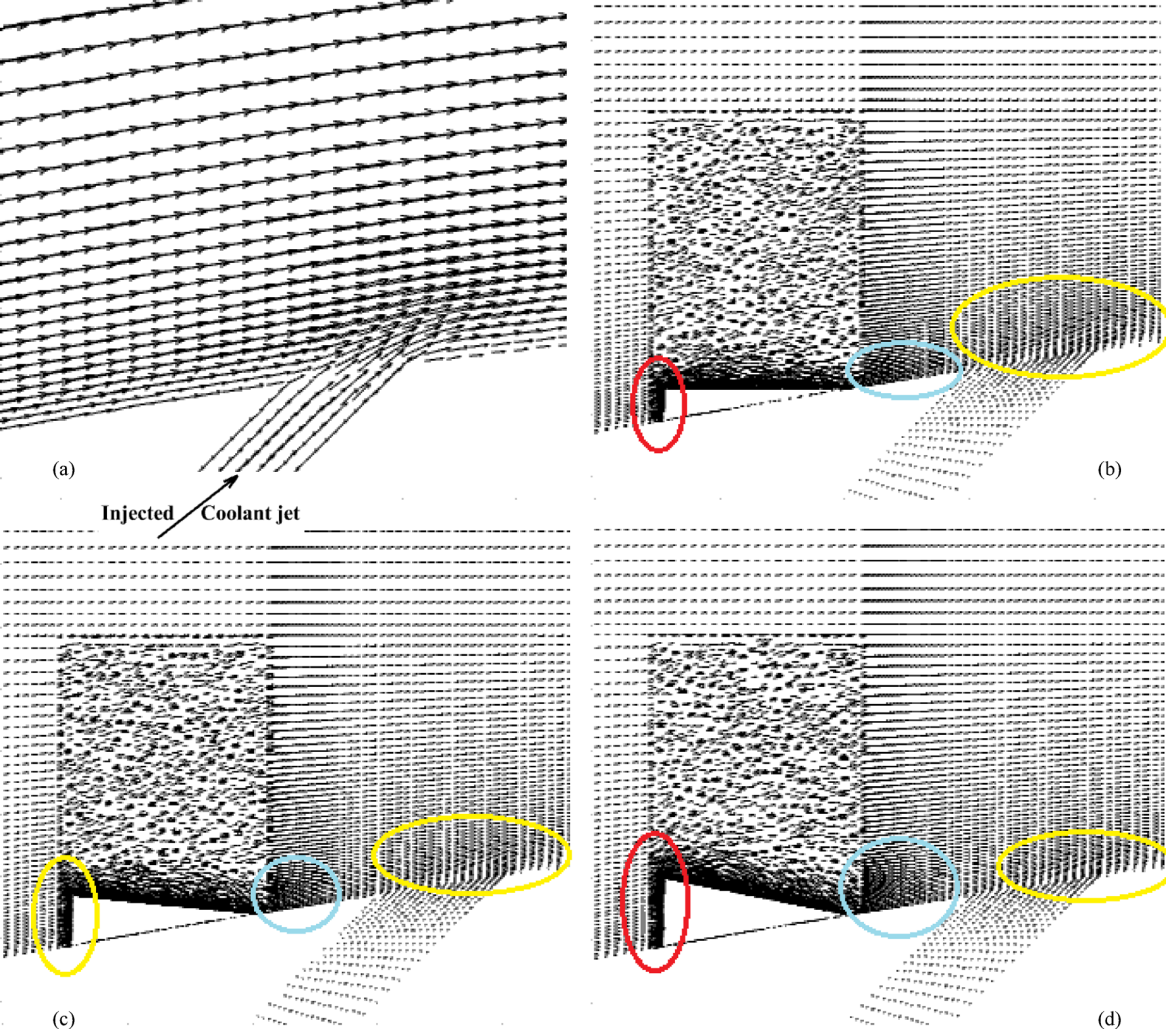
Mixing dynamics and flow field variations around the cooling hole and MVG. *X*-velocity vectors on the stator blade after film cooling: (**a**) without MVG; and with MVGs at heights of (**b**) 2.5 mm; (**c**) 4 mm; and (**d**) 5.5 mm.

**Fig. 12:**
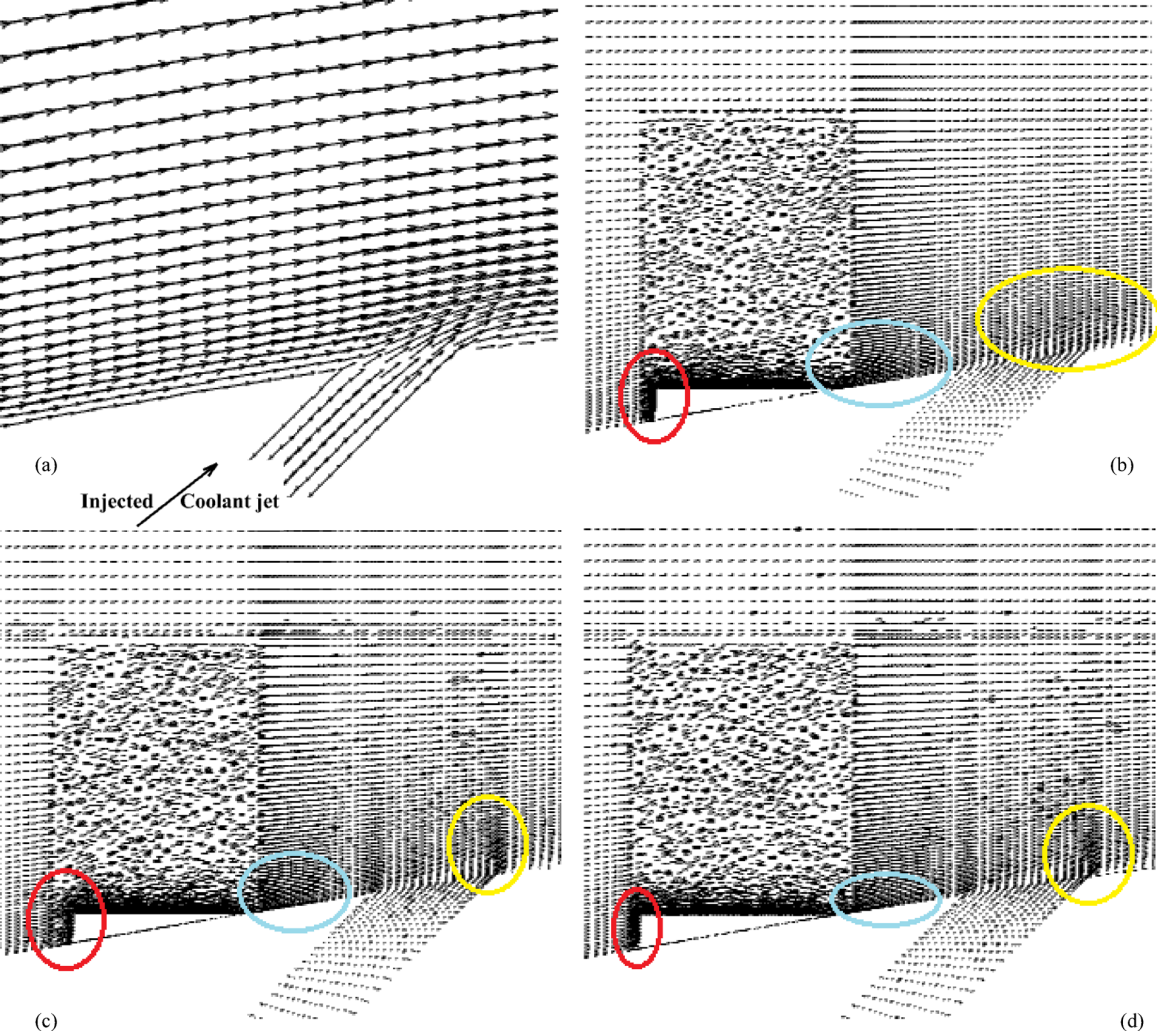
Mixing dynamics and flow field variations around the cooling hole and MVG. *X*-velocity vectors on the stator blade after film cooling: (**a**) without MVG, and with MVGs; (**b**) at the initial position; (**c**) − 5° apex angle; and (d) + 5° apex angle.

**Fig. 13:**
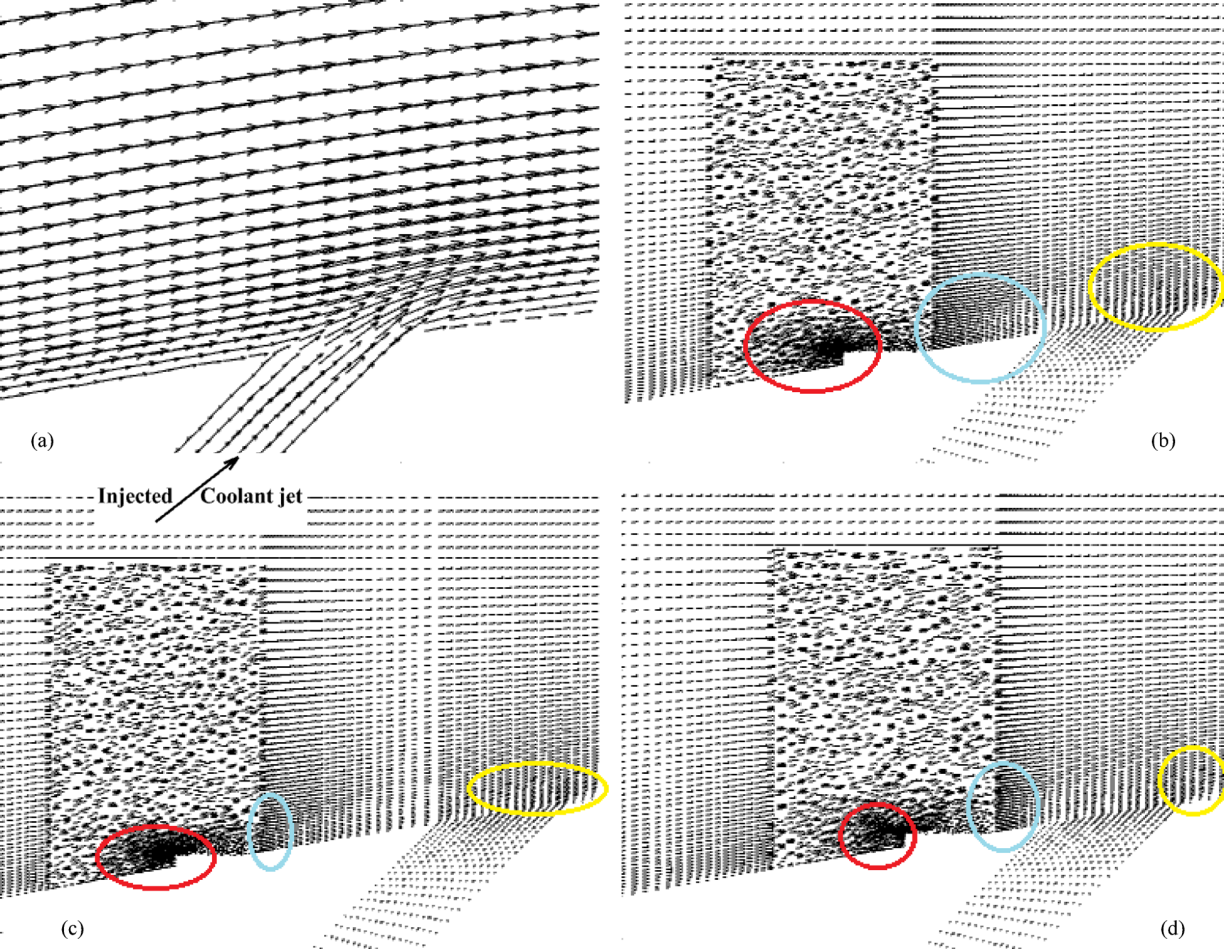
Mixing dynamics and flow field variations around the cooling hole and MVG. *X*-velocity vectors on the stator blade after film cooling: (**a**) without MVG, and with MVGs; (**b**) at the initial position; (**c**) + 5 mm from the injection point; and (**d**) − 5 mm closer to the injection point.

To facilitate a clearer interpretation of the flow behavior, the vector plots in Figs. 11, 12 and 13 illustrate the flow near the injection of the cooling jet into the hot transient flow over the blade and the MVG. Characteristic locations derived from the TKE distributions (Figs. 19, 20 and 21) and turbulent viscosity distributions (Figs. 22, 23 and 24) were marked on these vector plots. These locations correspond to regions where turbulence intensity and momentum exchange are most pronounced, highlighting areas of strong mixing and momentum transfer between the coolant jet and the mainstream, and providing physical reference points for examining their interaction.

According to Fig. 11, in case (a), without the MVG, the cooling flow rapidly separates from the surface, leading to ineffective cooling, and the temperature distribution continuously increases along the surface. In case (b), with an MVG height of 2.5 mm, the placement of the MVG results in the greatest temperature reduction, and the spread of velocity vectors behind the MVG indicates that the cooling flow is well-distributed and mixes effectively with the mainstream flow. This appropriate mixing enhances heat transfer, leading to effective cooling. In case (c), when the MVG height increases to 4 mm, the temperature distribution resembles that of the no-MVG case, indicating that the presence of the MVG does not provide a significant improvement in cooling performance. The minimal spread of velocity vectors behind the MVG in this case suggests that the cooling flow is not adequately distributed, reducing its mixing with the mainstream flow. Although there is slightly greater rotation and density above the MVG than in case (a), this effect is insufficient to enhance cooling. In case (d), with a height of 5.5 mm, the MVGs hinder cooling, leading to temperatures even higher than in the no-MVG case. The increased density of velocity vectors behind the MVG compared to case (b), along with the highest rotation and density above the MVG, indicates the formation of stronger vortices in this scenario. This performance drop is attributed to “disturbance in the boundary layer,” as taller MVGs generate stronger vortices that can cause excessive turbulence and instability in the cooling film, reducing heat transfer efficiency. This disturbance is more pronounced at 5.5 mm, resulting in higher surface temperatures. Therefore, the optimal height is 2.5 mm (case b), which yields the greatest temperature reduction. Exceeding this height (to 4 mm and 5.5 mm) can lead to reduced or even negative effects on cooling. The appropriate height of the MVG is crucial for achieving optimal mixing and preventing disturbances in the boundary layer.

The negative value of the Nusselt number (*Nu*) in areas where the MVG is located can be attributed to several factors. First, the presence of the MVG may cause flow separation, thereby reducing the effective heat transfer area. When flow detaches from the surface, it creates regions where heat transfer decreases significantly, resulting in negative *values of the Nusselt number*. Additionally, the increased turbulence generated by the MVG may enhance heat transfer in some areas; however, this effect may not be sufficient to overcome the cooling effect of the injected jet in other regions. If the cooling effect is stronger than the heat transfer enhancement, it can lead to lower or negative *Nu* values. Lastly, the interaction between the hot airflow and the cooling jet can alter the thermal boundary layer, leading to localized cooling effects that may further contribute to negative Nusselt numbers in those areas.

According to Fig. 12, the film cooling performance without an MVG (case a) is ineffective, as the cooling flow separates from the surface quickly. In case (b), where the MVG is positioned at its initial configuration, the highest density and spread of velocity vectors are observed around and behind the MVG, indicating concentrated flow and the formation of strong vortices. In case (c), with a − 5° apex angle, the density of velocity vectors is slightly reduced compared to case (b). In contrast, higher-velocity vectors are observed in front of the MVG, suggesting accelerated flow or a different flow pattern in this region. In case (d), with a + 5° apex angle, the density of velocity vectors is even lower than in case (c). However, in both cases (c) and (d), concentrated velocity vectors are observed at the interaction point between the cooling jet and the hot mainstream flow over the stator blade, indicating enhanced flow mixing in this region. The presence of MVGs at all three configurations (b, c, and d) reduces the stator surface temperature by up to 20% compared to the case without MVGs (a). Importantly, variations in the MVG apex angle between + 5° and − 5° do not significantly affect cooling performance or surface temperature. This suggests that the primary improvement in cooling is due to the presence of the MVG itself, while minor angle changes have a negligible impact on performance.

Based on Fig. 13, in Case (a), film cooling without an MVG results in the cooling flow separating from the surface quickly, leading to reduced cooling performance. In Case (b), where the MVG is in the base position, the highest concentration of velocity vectors occurs around and behind the MVG, creating vortices that keep the cooling flow closer to the surface. This configuration shows the strongest flow focus and vortex formation, with the largest velocity magnitudes observed behind the MVG, enhancing cooling performance. In Case (c), where the MVG is shifted 5 mm downstream from the injection point, the velocity vectors around and behind the MVG are less concentrated than in Case (b). Still, the stronger vortices formed at this position optimize flow mixing and heat transfer, achieving better cooling performance compared to the base position. In Case (d), where the MVG is moved closer to the injection point, the velocity vectors around the MVG are the least concentrated, and cooling performance decreases compared to Cases (b) and (c). The proximity of the MVG to the injection disrupts the flow, hindering proper distribution of the cooling air. Despite this, the velocity vectors behind the MVG are more concentrated than in Case (c), indicating that the flow re-focuses after passing the MVG. However, the overall cooling performance remains weaker. These findings highlight the significant impact of MVG positioning on cooling performance. In contrast, all MVG configurations (b, c, d) improve cooling compared to Case (a), positioning the MVG 5 mm downstream (Case c) provides the best results by optimizing flow mixing and heat transfer. Conversely, positioning the MVG too close to the injection point (Case d) disrupts the flow and reduces cooling efficiency.

**Fig. 14:**
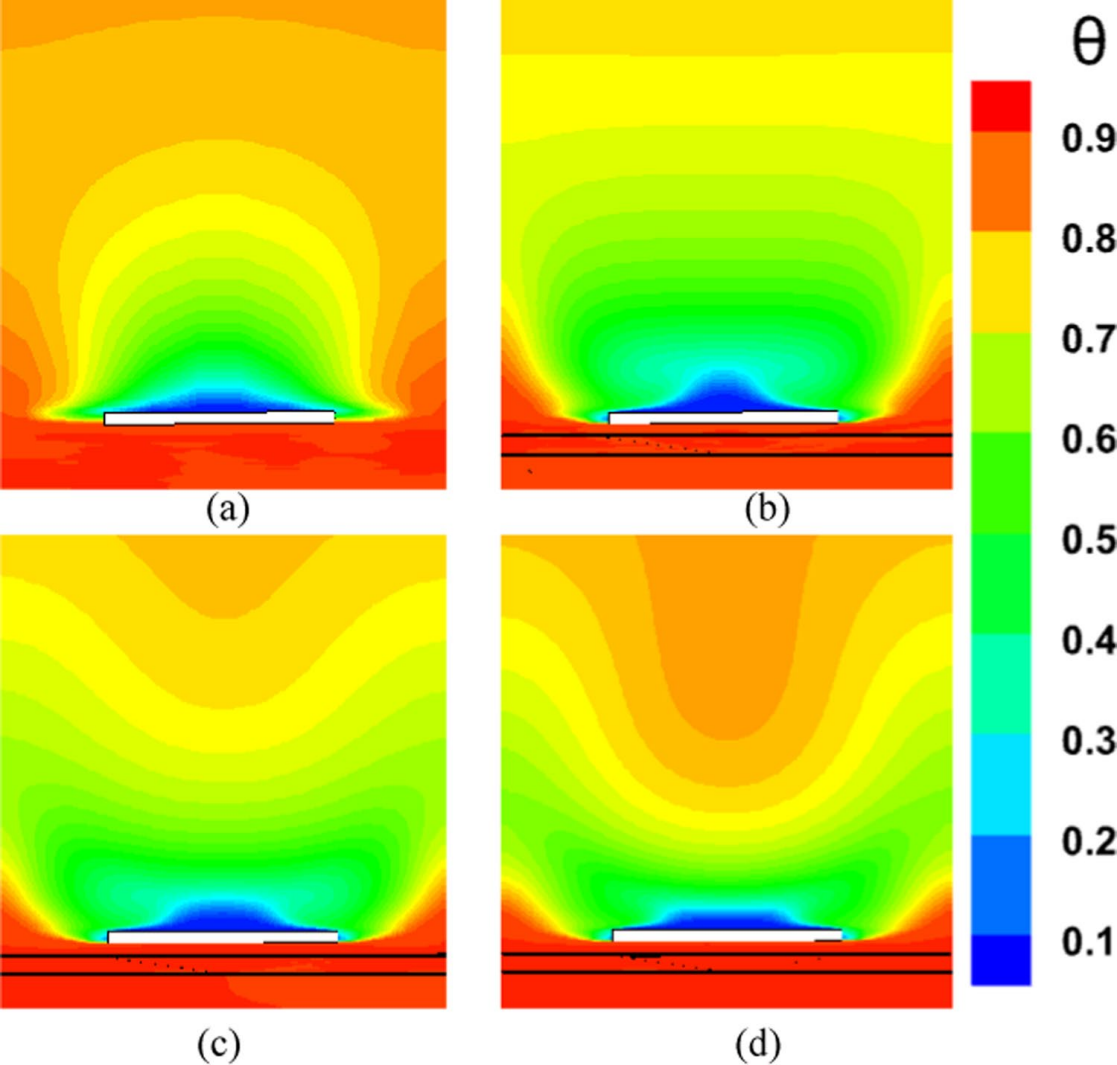
Surface temperature contours after film cooling: (**a**) without MVG and with MVGs at heights of (**b**) 2.5 mm; (**c**) 4 mm; and (**d**) 5.5 mm.

As shown in Fig. 14, by introducing controlled turbulence into the flow, MVGs prevent the cooling jet from concentrating in localized areas and instead distribute it more uniformly across the blade. This uniform distribution reduces the occurrence of hot spots and ensures broader surface coverage.. In addition to lateral spreading, MVGs influence the longitudinal propagation of the cooling jet along the blade. By inducing greater mixing within the flow, MVGs help retain the jet’s kinetic energy, enabling it to penetrate further along the blade surface. This effect is particularly evident when MVGs are set to an optimal height, such as 2.5 mm in this study. At this height, MVGs generate appropriately sized vortices that enhance lateral spreading while maintaining the jet’s velocity and penetration depth. As a result, cooling efficiency is maximized both across the blade span and along its length. However, when the MVG height exceeds the optimal range, a different dynamic is observed. Although lateral spreading still occurs, larger MVGs generate stronger and more complex vortex structures. These include intense vortices and counter-rotating vortex pairs that introduce excessive turbulence into the flow. The heightened turbulence not only scatters the cooling jet more widely but also creates significant resistance to the jet’s forward motion. Consequently, the kinetic energy of the cooling jet is consumed maintaining these complex vortex structures rather than advancing along the blade. This leads to a noticeable reduction in the cooling jet’s penetration depth and longitudinal effectiveness. while an optimal MVG height improves both lateral and longitudinal cooling performance, excessive heights compromise the cooling jet’s ability to propagate along the blade by introducing excessive turbulence. Achieving the ideal balance ensures the cooling jet is effectively distributed and penetrates the blade surface to its maximum potential, minimizing hot spots and improving overall thermal management.

**Fig. 15:**
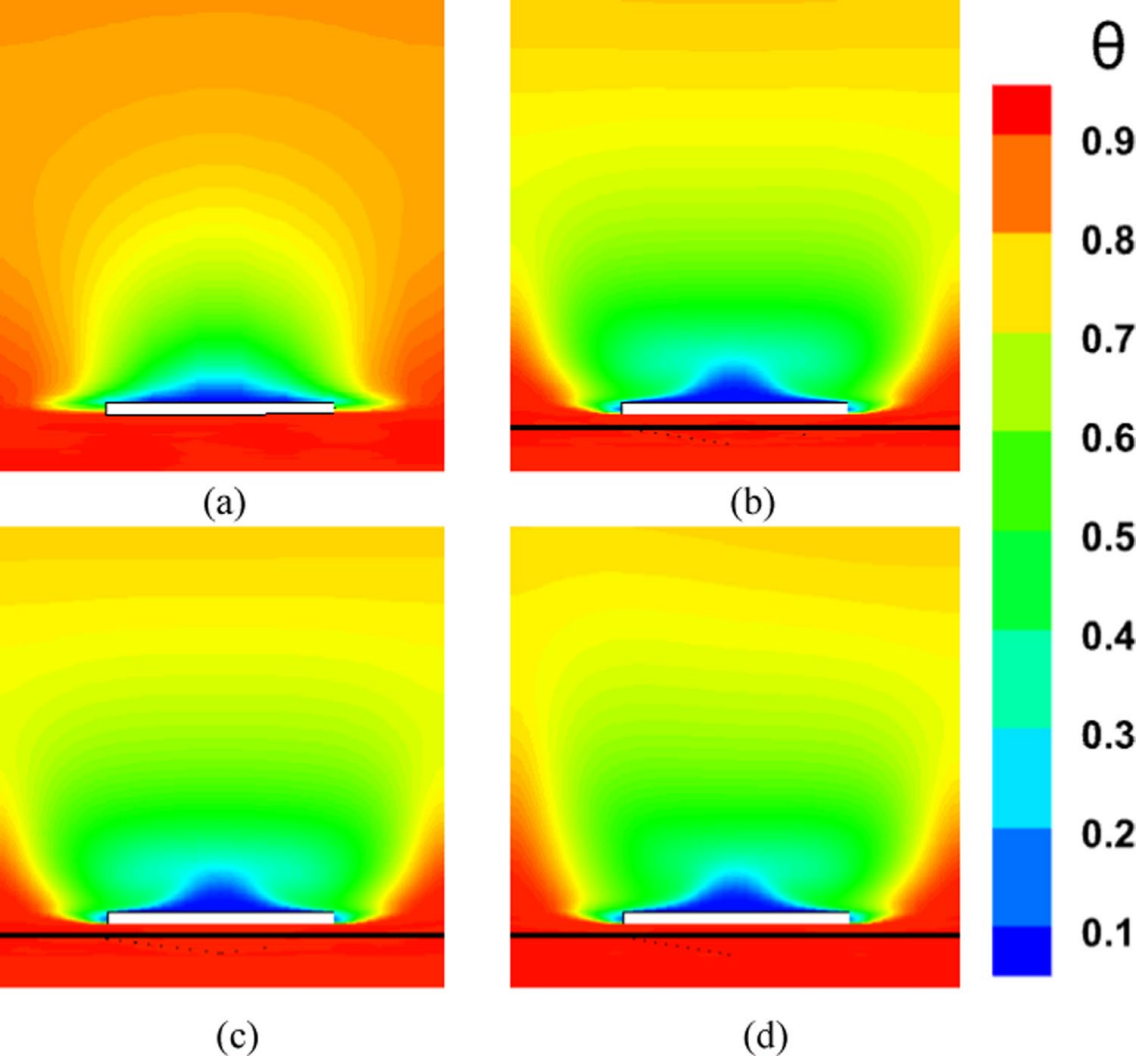
Surface temperature contours after film cooling: (**a**) without MVG; (**b**) at the initial MVG position; (**c**) with − 5° apex angle; and (**d**) with + 5° apex angle.

**Fig. 16:**
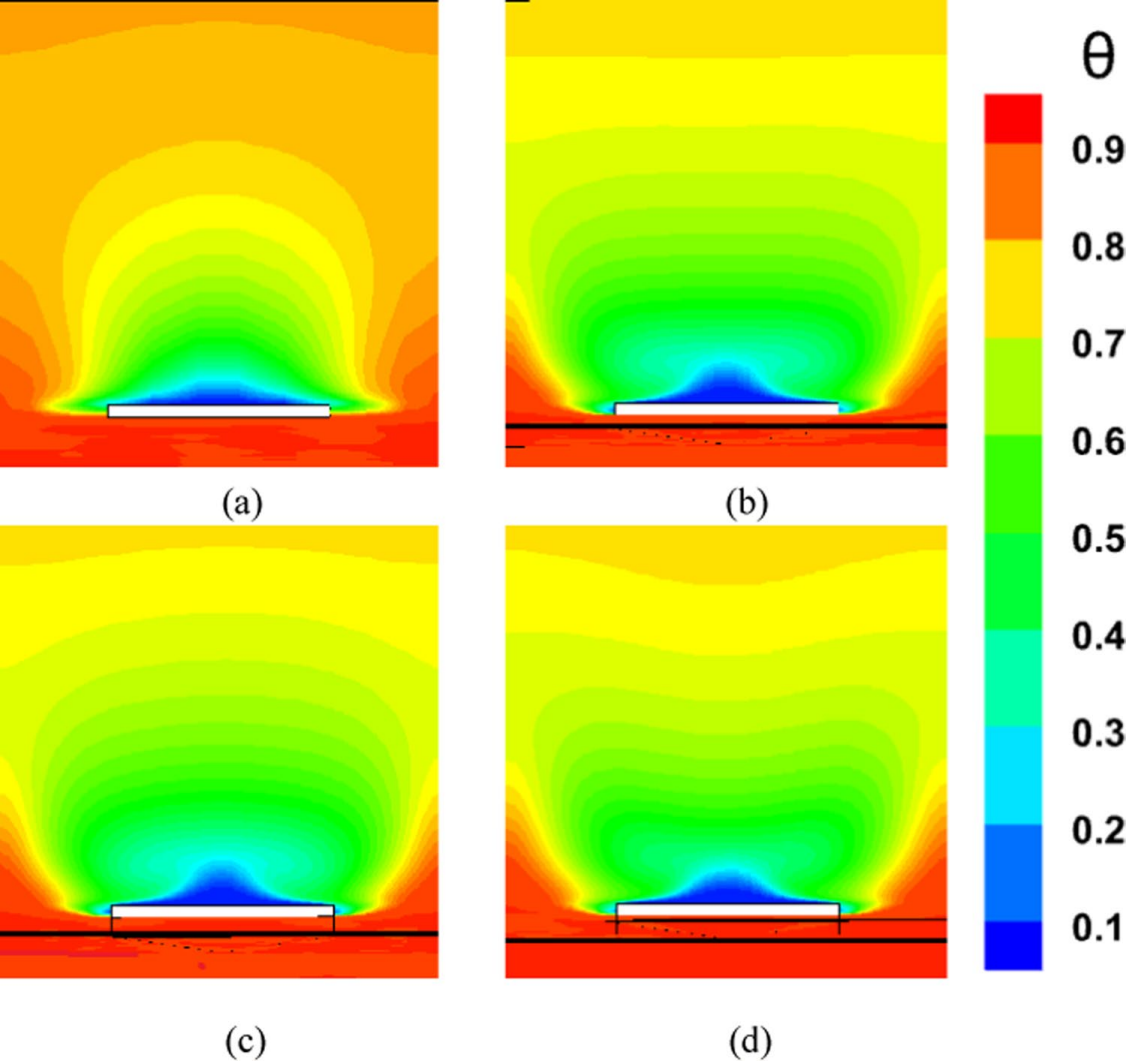
Surface temperature contours after film cooling: (**a**) without MVG; (**b**) at the initial MVG position; (**c**) + 5 mm from the injection point; and (**d**) − 5 mm closer to the injection point.

**Fig. 17:**
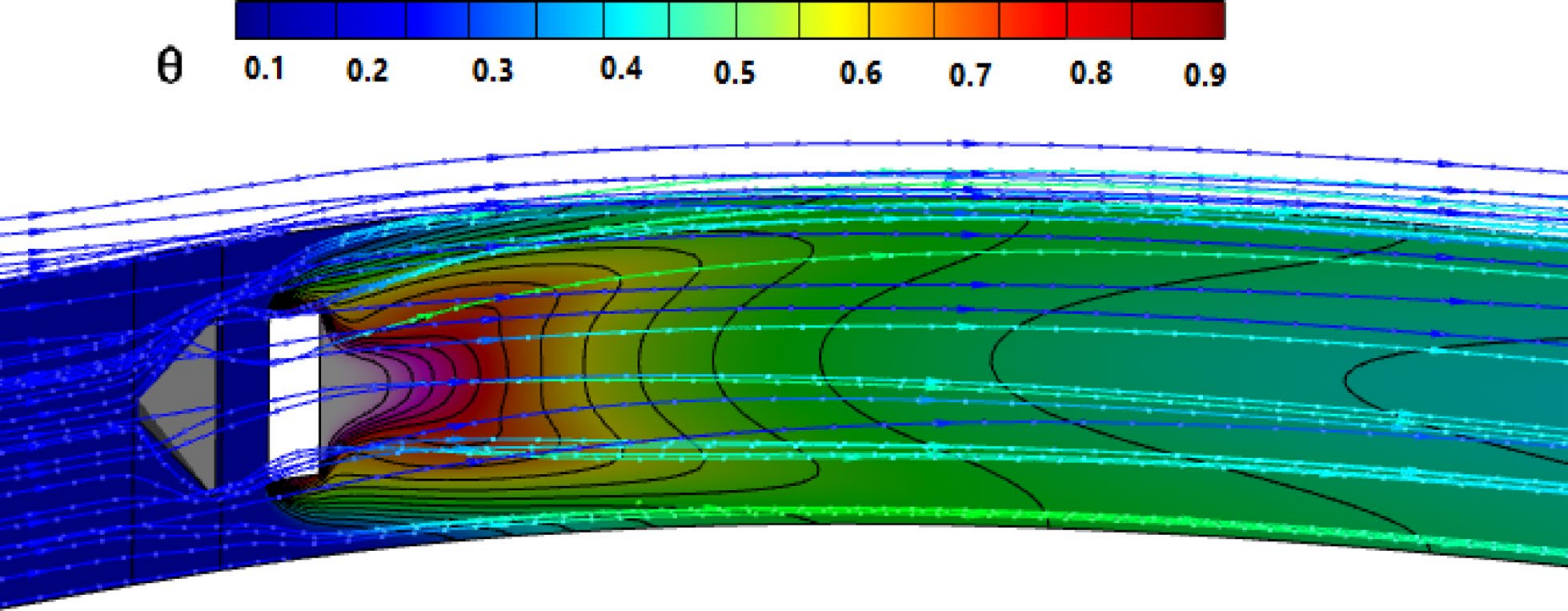
Contours of streamlines for hot airflow and cooling jet around the MVG and blade holes.

**Fig. 18:**
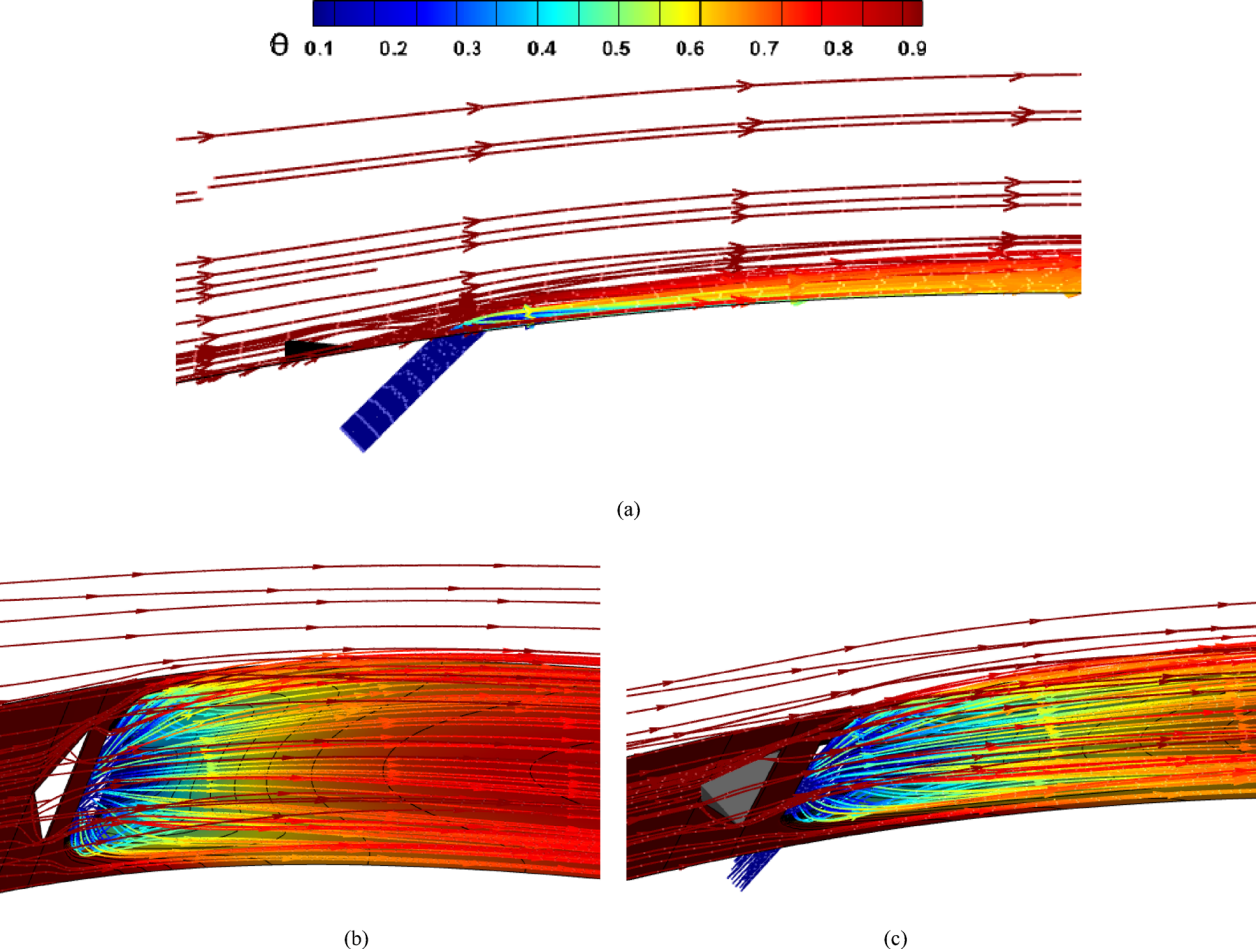
(**a**) Flow patterns surrounding the cooling hole and MVG; (**b**, **c**) interaction and mixing of hot and cold flows.

In Figs. 14, 15, 16, 17 and 18, the temperature contours display the non-dimensional temperature, θ, which is normalized so that the minimum and maximum correspond to a ratio of 1:2, covering the actual range of 300–600 K, as indicated in the legend.

Figure 15 shows that altering the MVG angle has little impact on the longitudinal cooling distribution along the blade. In case (c), with a − 5*°* apex angle, the cooling jet spreads more effectively across the blade, improving lateral cooling. Conversely, in case (d), where the MVG is positioned 5 mm closer to the injection point, the lateral spread of the cooling jet is at its minimum.

According to Fig. 16, the cooling jet shows a greater lateral spread in case (d), where the MVG is positioned − 5 mm closer to the injection point. In contrast, case (c), with the MVG + 5 mm from the injection point, exhibits a more concentrated cooling effect along the blade’s centerline. However, the presence of the MVG significantly improves both the lateral distribution of cooling and its penetration into the blade’s longitudinal depth.

The streamline visualizations in Figs. 17 and 18 illustrate the flow behavior around the blade and the MVG, as well as the interaction between the injected cooling jet and the hot mainstream flow. As the hot flow encounters the MVG, it is redirected, forming a pair of counter-rotating vortices downstream. These vortices enhance mixing within the boundary layer, altering both its velocity and thermal profiles. In this region, localized flow separation occurs, creating a low-pressure recirculation zone that influences heat transfer. The streamlines of the injected cooling jet indicate that, after exiting the groove, the jet is influenced by the MVG-generated vortices, causing its trajectory to shift toward low-pressure regions. This interaction enhances mixing between the cooling jet and the hot flow, leading to a more dispersed jet and increased surface coverage. Compared to the case without an MVG, the presence of the vortex generator increases turbulence, improving heat transfer in the injection region. As a result, the blade’s cooling effectiveness and overall heat transfer characteristics are modified.

**Fig. 19:**
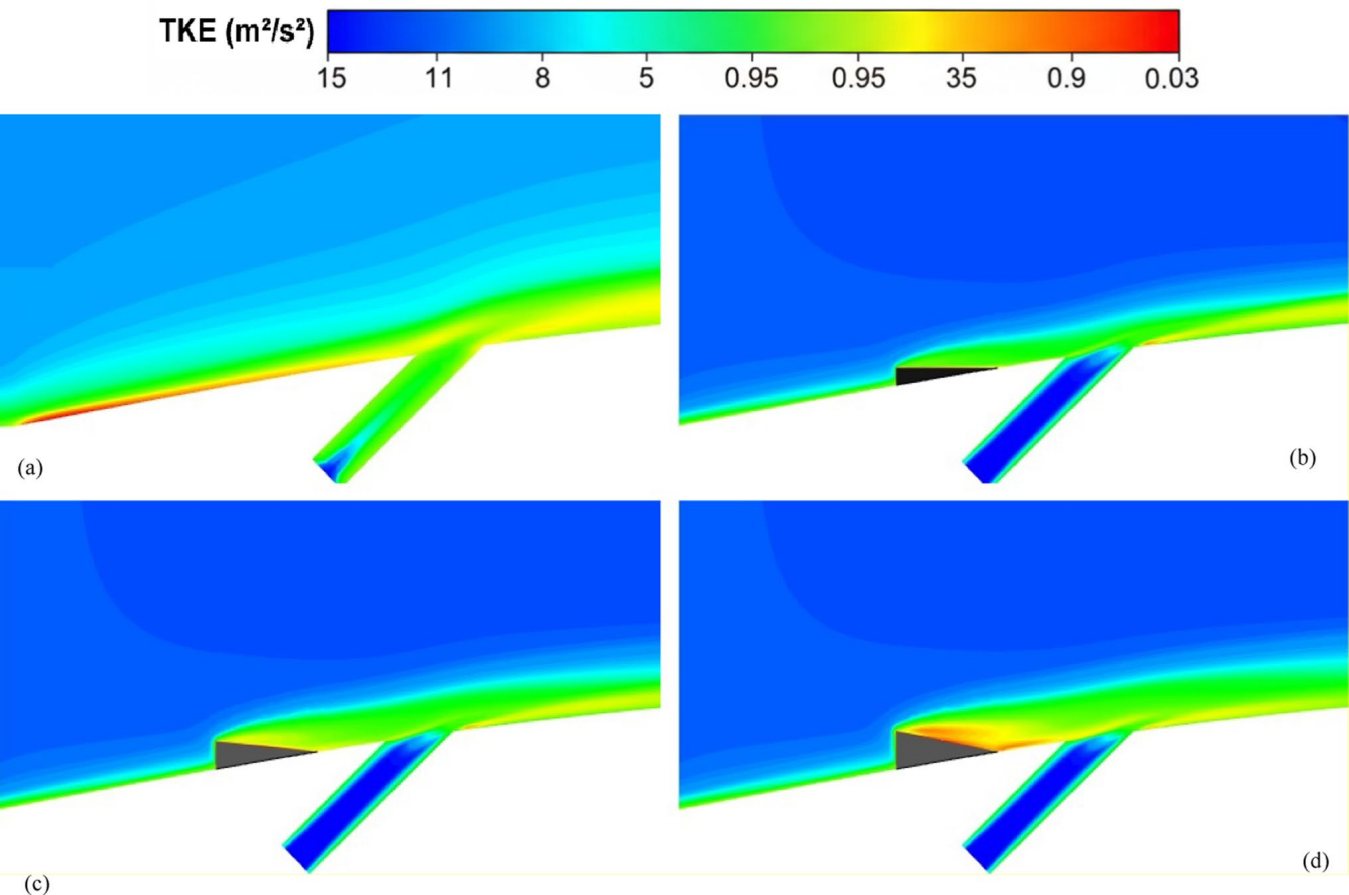
Turbulence kinetic energy (TKE) contours after film cooling: (**a**) without MVG and with MVGs at heights of (**b**) 2.5 mm; (**c**) 4 mm; and (**d**) 5.5 mm.

**Fig. 20:**
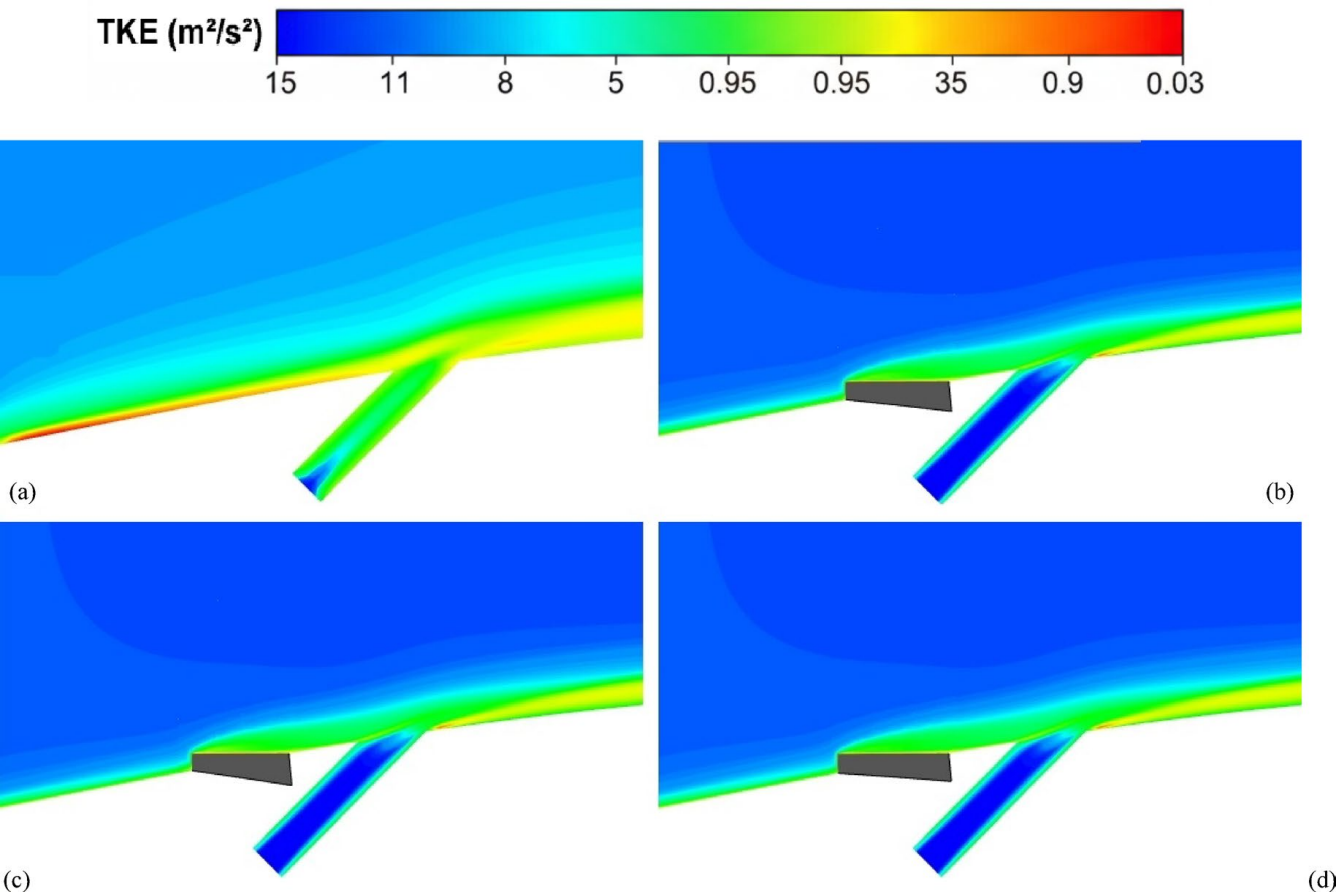
Turbulence kinetic energy (TKE) contours after film cooling: (**a**) without MVG; (**b**) at the initial MVG position; (**c**) with − 5° apex angle; and (**d**) with + 5° apex angle.

**Fig. 21:**
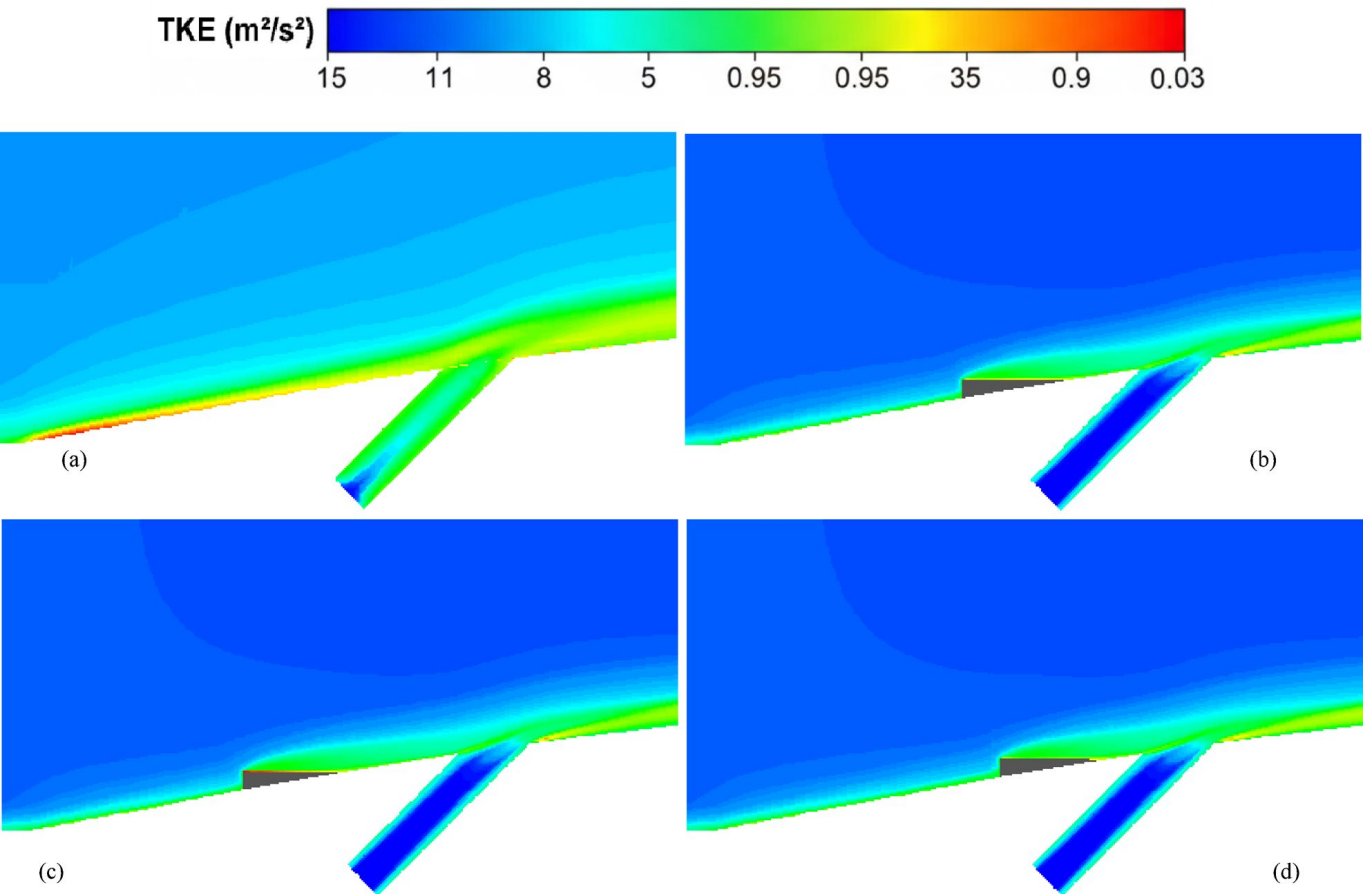
Turbulence kinetic energy (TKE) contours after film cooling: (**a**) without MVG; (**b**) at the initial MVG position; (**c**) + 5 mm from the injection point; and (**d**) − 5 mm closer to the injection point.

**Fig. 22:**
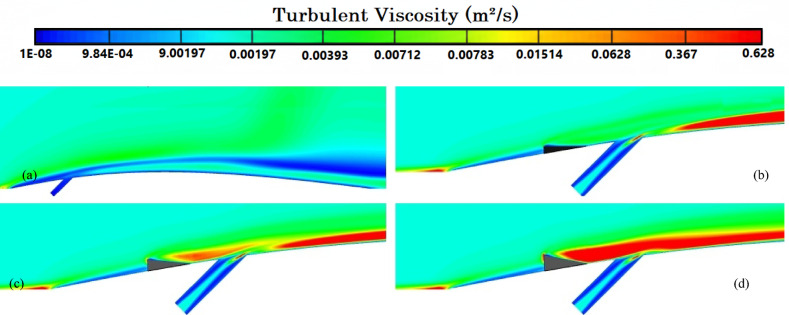
Turbulence viscosity contours after film cooling: (**a**) without MVG and with MVGs at heights of (**b**) 2.5 mm; (**c**) 4 mm; and (**d**) 5.5 mm.

**Fig. 23:**
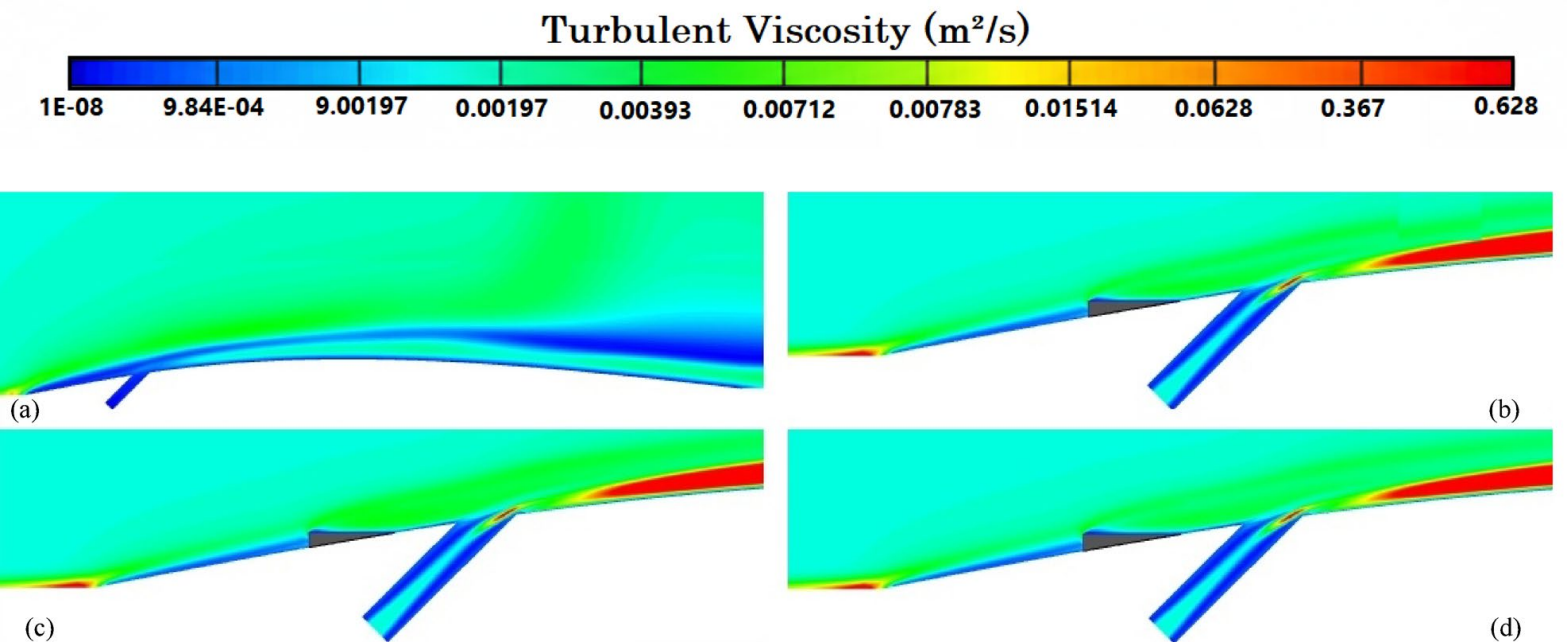
Turbulence viscosity contours after film cooling: (**a**) without MVG; (**b**) at the initial MVG position; (**c**) with − 5° apex angle; and (**d**) with + 5° apex angle.

**Fig. 24:**
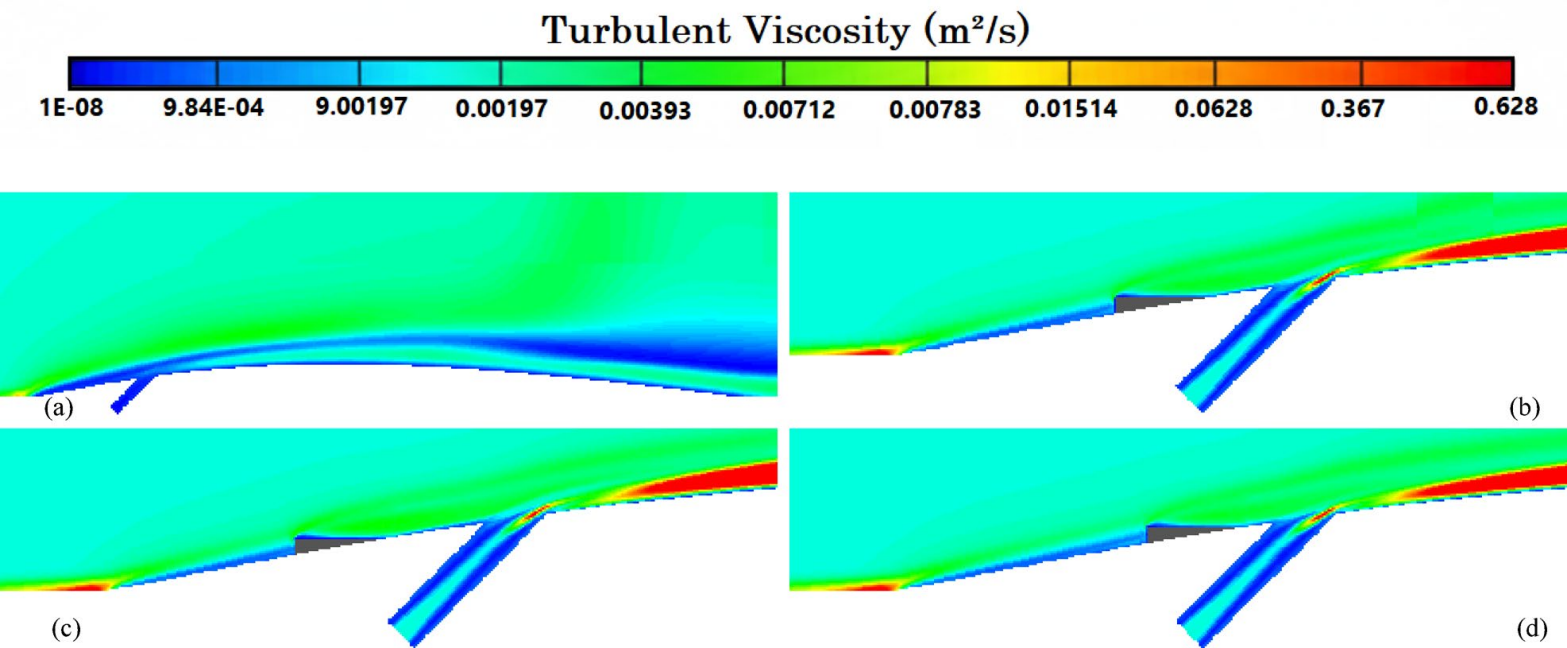
Turbulence viscosity contours after film cooling: (**a**) without MVG; (**b**) at the initial MVG position; (**c**) + 5 mm from the injection point; and (**d**) − 5 mm closer to the injection point.

The turbulent kinetic energy (TKE) contours (Figs. 19, 20 and 21) and turbulent viscosity contours (Figs. 22, 23 and 24) are included to illustrate the characteristics of turbulence and their impact on surface cooling. TKE represents the intensity of velocity fluctuations and identifies regions where kinetic energy is available to enhance mixing between the cooling jet and the main flow. Higher TKE regions correspond to stronger interactions and more effective dispersion of the coolant. Turbulent Viscosity indicates the local momentum exchange caused by turbulence, showing how the flow spreads laterally, penetrates near the surface, and distributes the cooling effect. Together, these contours reveal how the cooling jet interacts with the main flow and nearby vortices, highlighting areas where turbulence promotes near-wall mixing and affects the distribution of the coolant along the blade surface.

As illustrated in Fig. 19, increasing the height of the micro vortex generator (MVG) intensifies the streamwise vortices and strengthens the near-wall TKE in the jet–crossflow interaction zone. Compared with the no-MVG case, all heights suppress the large TKE pocket associated with recirculation at the hole exit and promote a more organized turbulent structure downstream. The 2.5 mm MVG produces coherent vortices that energize the boundary layer without excessive mixing, maintaining a compact TKE streak close to the wall. At 4.0 mm, the high-TKE region broadens, enhancing attachment but extending the mixing zone into the core. When the height reaches 5.5 mm, the TKE layer thickens considerably, suggesting stronger but more intrusive mixing. Overall, increasing height improves turbulence organization up to 4.0 mm, beyond which over-mixing may deteriorate film stability and increase drag.

Figure 20 shows that all apex-angle configurations produce similar TKE structures, characterized by paired streamwise vortices and a near-wall layer stabilized by the apex angle. Small angular deviations of ± 5° shift the downwash location slightly but do not significantly alter the magnitude or footprint of the TKE field. This indicates that within this range, the vortex coherence and wall energization remain largely unaffected by the apex angle. Therefore, small variations in apex angle act as a secondary tuning parameter, with minimal influence compared to MVG height or position. As seen in Fig. 21, moving the MVG 5 mm downstream aligns the high-TKE region more effectively with the jet shear layer, sustaining organized turbulence over a longer distance. Conversely, positioning the MVG 5 mm upstream initiates earlier vortex formation, but the TKE decays before reaching the hole-exit region. Both configurations outperform the no-MVG case by reducing detached TKE pockets near the injection slot. The results suggest that the streamwise position strongly influences the synchronization between vortex generation and jet shear. A modest downstream shift (+ 5 mm) yields the most persistent near-wall TKE and enhances film attachment.

Figure 22 displays the turbulent viscosity contours for various MVG heights. The introduction of the MVG markedly increases μₜ in the jet–crossflow region, forming a high-μₜ layer that closely follows the coolant film along the wall. As the MVG height increases, this layer thickens and extends farther into the mainstream. At 2.5 mm, μₜ remains concentrated near the wall, supporting momentum exchange while maintaining film coherence. Increasing the height to 4.0 mm enhances lateral mixing, although some of the turbulence extends upward into the core. At 5.5 mm, μₜ spreads excessively, indicating over-mixing that can weaken film uniformity. Hence, an MVG height of 2.5–4.0 mm provides the best balance between turbulence intensity and film stability.

As illustrated in Fig. 23, all apex angles yield nearly identical μₜ distributions associated with the MVG-induced vortex pair. Minor angular adjustments (± 5°) slightly tilt the high-μₜ regions without significantly altering their intensity or extent. This consistency implies that the turbulent viscosity field depends primarily on vortex strength and height rather than on small changes in apex angle. Therefore, within this range, the apex angle has a limited effect on enhancing near-wall mixing or film attachment.

Figure 24 compares the μₜ fields for different streamwise positions of the MVG. A 5 mm downstream shift produces a continuous high-μₜ path along the wall that overlaps with the jet’s shear layer, indicating sustained turbulence where it benefits film adhesion. In contrast, placing the MVG 5 mm upstream results in μₜ being earlier, but leaves a gap near the injection zone, indicating weaker interaction with the jet core. These findings demonstrate that the streamwise position determines the phase-matching between the vortex pair and the coolant jet. A moderate downstream relocation (+ 5 mm) most effectively enhances near-wall turbulent mixing while minimizing unnecessary turbulence farther from the wall.

The collective results confirm that MVG height is the dominant factor controlling vortex strength, TKE, and μₜ distribution, with 2.5–4.0 mm offering optimal film stability and mixing. The apex angle (± 5°) plays only a minor role, while streamwise position affects the phase alignment between vortex motion and jet development. A slight downstream offset provides the most coherent and wall-attached turbulence structure, leading to improved coolant coverage and reduced jet lift-off. In summary, it is essential to prioritize height and position to place strong—but wall-focused—turbulence where it anchors the film without eroding it.

## Conclusion

This study comprehensively evaluated the impact of MVGs on both aerodynamic and thermal performance in stator blade cooling. Several configurations were examined, varying in MVG height, streamwise position, and apex angle. Key parameters assessed included turbulence kinetic energy (TKE), surface pressure, wall temperature, film cooling effectiveness, enthalpy, stagnation density, velocity magnitude, wall shear (cfRe), and the Nusselt number.

The results show that MVGs can effectively enhance coolant coverage and convective heat transfer by modifying the boundary layer structure and improving the interaction between the coolant jet and the mainstream hot flow. Specifically, streamwise vortices generated by MVGs energize the near-wall region, promoting earlier flow reattachment and increasing thermal mixing. These effects were most pronounced when the MVG was placed 5–7.5 mm upstream of the injection hole, with heights between 2.5 and 4.0 mm. Within this configuration, cooling performance improved by 33–46% compared to the baseline, particularly over the root and mid-chord regions (X/D ≈ 0.05–0.2), where the vortices help maintain a coherent and attached cooling film. Among all tested cases, the 2.5 mm height MVG achieved the highest temperature reduction of 13.6%. Increasing the height to 4 mm provided no additional benefit, while the 5.5 mm case worsened performance, with a 10.23% rise in wall temperature. Streamwise location also played a crucial role: moving the MVG 5 mm downstream increased efficiency by 16%, whereas placing it 5 mm upstream yielded only a 5% improvement. Apex angle variations (± 5°) had a minimal effect on thermal and flow fields, indicating that small angular changes are a secondary design factor under these conditions.

Aerodynamically, MVGs promoted earlier boundary layer reattachment, reduced TKE peaks, and increased surface pressure recovery. Thermally, they enhanced Nusselt numbers through improved jet–mainstream mixing; however, excessive vortex strength led to early turbulence decay and reduced downstream thermal performance. Overall, properly configured MVGs can significantly improve internal cooling, with height and streamwise position being the most influential parameters. When tuned carefully, MVGs enhance thermal uniformity, strengthen surface protection, and maintain favorable flow characteristics, making them a valuable passive solution in high-speed cooling system design.

It should be noted that this study is limited to steady-state simulations of jet impingement cooling in microchannels, assuming ideal boundary conditions and a single-phase, single-species gas. Transient effects, real gas behavior, and multi-species or multiphase interactions were not considered. The blade geometry was simplified, excluding detailed curvature and surface roughness, to focus on the fundamental mechanisms governing flow and heat transfer under the studied conditions. In real turbine environments, the flow would be influenced by thermal gradients, unsteady fluctuations, and rotational effects. Future studies will extend this work by considering more realistic operating conditions and exploring a broader range of design parameters.

## Data Availability

The datasets used and/or analyzed during the current study are available from the corresponding author upon reasonable request.
